# Rhizosphere microecological characteristics associated with tobacco root-knot nematode disease

**DOI:** 10.3389/fmicb.2026.1874628

**Published:** 2026-07-16

**Authors:** Tingting Lu, Xuanquan Zhu, Meng Jia, Enxing Li, Jie Li, Zhengling Liu, Duo Wang, Qionghua Chen, Runling Wang, Peng Zhou, Yu Du, Qi Li, Na Wang, Ge Wang, Yuxiang Bai

**Affiliations:** 1 Yunnan Agricultural University, Kunming, Yunnan, China; 2 The Key Laboratory of Agricultural Microbiome of Yunnan Province, Kunming, Yunnan, China; 3 Yunnan Tobacco Company Yuxi City Company, Yuxi, Yunnan, China; 4 Yunnan Tobacco Company Kunming City Company, Kunming, Yunnan, China; 5 Yunnan Tobacco Quality Supervision and Testing Station, Kunming, Yunnan, China

**Keywords:** microbial community, root-knot nematode, soil chemistry and physics, soil enzyme activity, tobacco

## Abstract

Root-knot nematode disease poses a serious threat to the production of tobacco and other crops, but the rhizosphere microecological response mechanisms during disease development remain unclear. In this study, we collected rhizosphere soil from healthy and diseased tobacco plants in typical tobacco-growing areas of Kunming, Yunnan Province, China, and we systematically identified the differences in soil physicochemical properties, enzyme activities, and the microbial community composition and structure based on 16S rDNA and ITS amplicon sequencing. Compared to healthy rhizosphere soil, diseased rhizosphere soil showed significant decreases in pH (from 5.39 ± 0.37 to 4.98 ± 0.23), catalase (CAT) activity (23.89% reduction), and the relative abundance of health-associated microbial taxa, particularly bacterial species *Flavisolibacter ginsengisoli* (71.05% reduction) and fungal species *Fusicolla acetilerea* (87.96% reduction). In addition, bacterial communities in diseased soil exhibited a predicted enrichment of functional pathways related to metabolism, environmental adaptation, and stress response (e.g., ansamycin biosynthesis and bacterial chemotaxis). Associations among these observed changes may reflect a cycle potentially linked to disease development. Conversely, healthy soils showed a higher pH, CAT activity, and relative abundance of health-associated microbial taxa. Together, these results reveal the coordinated changes among soil physicochemical factors, enzyme activities, and microbial communities and suggest an association between rhizosphere microecological imbalance and root-knot nematode disease. These findings provide a theoretical basis for future studies exploring the targeted regulation of soil microecology for managing this disease.

## Graphical abstract

**Figure:**
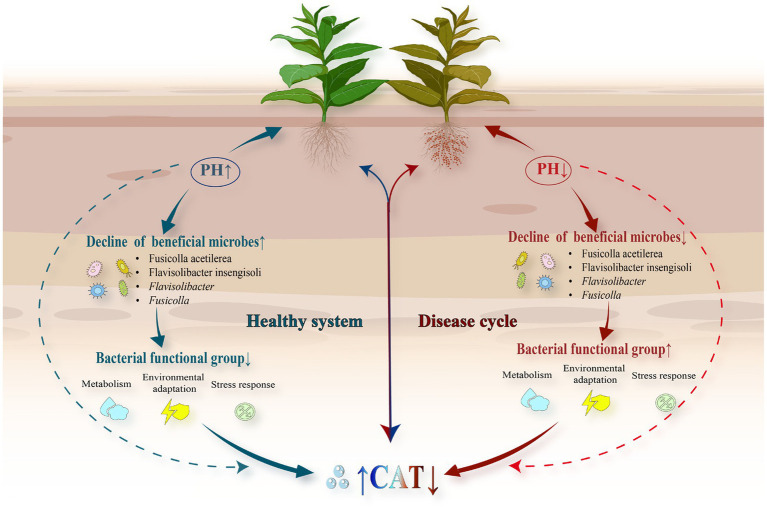

## 1 Introduction

Root-knot nematode (RKN) disease is a major soil-borne disease caused by RKNs (*Meloidogyne* spp.) infecting plant roots. It affects a wide range of crops and leads to significant yield losses (Cao et al., 2023; Sang et al., 2024). Approximately 100 species of RKNs have been reported worldwide (Vos et al., 1998). They possess a wide host range, diverse reproductive strategies, and rapid transmission rates, and they are commonly found in industrial crops, cereals, ornamental plants, vegetables, and weeds (Vos et al., 1998). RKN disease is also a major tobacco disease. It has occurred in multiple tobacco-growing regions across China, including Northeast China (Heilongjiang, Liaoning), Central China (Henan, Hubei), and Southwest China (Yunnan, Sichuan), with the severity of damage continuously increasing (Zebin et al., 2015; Huang et al., 2020). Among these regions, the affected area in Yunnan Province exceeded 26,000 hectares, resulting in a yield loss of 30–50% (Zebin et al., 2015). Nematodes can infect tobacco roots at all of the growth stages, forming tumorous galls that damage the root vascular system and impede water and nutrient absorption and transport (Back et al., 2002). This leads to stunted plant growth, abnormal leaf development and deformation, and aberrant root systems; in severe cases, the entire plant wilts (Xu et al., 2023; Yadav et al., 2025). RKN infection directly damages tobacco and also facilitates co-infection by other pathogens, thus exacerbating the damage and severely restricting tobacco yield and quality.

The prevention and control of RKN is a major challenge in agricultural production. Currently, the main prevention and control strategies include biological, physical, chemical and agricultural methods. Among them, physical and agricultural control methods are economically and environmentally friendly, but they take longer to take effect and are greatly influenced by the environment; chemical control methods are quick to produce results and easy to operate, and they are currently the main approach, but they can easily lead to nematode resistance, environmental pollution and pesticide residues; biological control methods, due to their safety and practicality, are considered a better choice (Aioub et al., 2022). Among them, microbial control has the characteristics of high efficiency, green and sustainable development, and has great potential for development (Wang et al., 2022; Sang et al., 2024).

The rhizosphere soil is the narrow zone most strongly influenced by plant roots. It is rich in nutrients and serves as a hotspot for microbial interactions, harboring beneficial microorganisms, soil-borne pathogens, and their competing communities (Liu et al., 2016). Soil microorganisms are involved in the decomposition of organic matter and nutrient cycling, and they are closely linked to plant growth and disease spread (Chen et al., 2019; Zhou et al., 2023; Sang et al., 2024). The composition and diversity of microbial communities directly affect the occurrence of soil-borne diseases (Du et al., 2021; Tong et al., 2021). For example, beneficial microorganisms, such as *Bacillus*, *Pseudomonas*, and *Paenibacillus*, can antagonize pathogens (Wang et al., 2022), whereas microbial groups, including *Fusarium*, *Phytophthora*, and *Pythium*, may promote the development of certain soil-borne diseases (Liu et al., 2016).

Although biological control technology for tobacco RKN disease has made some progress, the efficacy of biological control remains unstable due to multiple factors, such as environmental conditions, microbial oxidative stress levels, and planting management systems (Du et al., 2021, Tong et al., 2021). A deeper understanding of the complex relationship between RKNs and various microorganisms is essential for developing sustainable and effective nematode management strategies (Rutter et al., 2022). Therefore, this paper analyzes the bacterial and fungal diversity, composition, structure, and interactions in the rhizosphere soil of diseased and healthy tobacco plants, with the aim of providing a theoretical basis for the efficient control of RKN.

## 2 Materials and methods

### 2.1 Soil sample collection

Based on the occurrence of tobacco RKN disease, soil samples were collected from three plots in which the disease was particularly severe within Kunming City, Fenghe Town, Xundian County (longitude: E103°4′0.48″, latitude: N25°51′2.88″, altitude: 2072 m); Aziying Township, Songming County (longitude: E102°49′59.52″, latitude: N25°19′30.72″, altitude: 2105 m); and Panlong District, Kunming City (longitude: E102°45′9.72″, latitude: N25°8′2.0394″, altitude: 1932 m). The soil at the sampling sites consisted entirely of red soil, characterized by flat terrain and moderate fertility. All of the sampling sites used traditional agricultural practices, including seedling cultivation, fertilization, pest control, and other field management techniques. As described in GB/T 23222—2008, diseased plants with disease grades 5–9 were selected (designated as D), and healthy plants (designated as H) 45 cm from the diseased plants were selected. Rhizosphere soil was collected using the shaking root method. Each sample was homogenized and divided into two portions: one frozen at −80 °C for DNA extraction and microbiome analysis, and the other used for soil physicochemical and enzyme activity assays. Three biological replicates were obtained from every sampling site, resulting in nine biological replicates per treatment.

### 2.2 Measurement of soil enzyme activities

The soil urease activity was determined using the phenol–sodium hypochlorite colorimetric method (Liu et al., 2021). The soil peroxidase activity was measured using the potassium permanganate titration method (Shi et al., 2023). The soil alkaline phosphatase activity was determined following the colorimetric method using sodium benzoate phosphate as the substrate (Liu et al., 2019). The soil sucrase activity was measured via the colorimetric method using 3,5-dinitrosalicylic acid (Saiya-Cork et al., 2002).

### 2.3 Determination of the physical and chemical parameters of soil samples

The soil pH was measured using a pH meter in a soil:water suspension at a ratio of 1:2.5 (w:v) (Xu et al., 2022). The soil organic matter (SOM) concentration was determined using the potassium dichromate oxidation–sulfuric acid titration method. Soil ammonium nitrogen (NH_4_^+^-N) and nitrate nitrogen (NO_3_^−^-N) were extracted with 1 mol/L potassium chloride solution in a soil:solution ratio of 1:2.5 (w:v). The NH_4_^+^-N content was determined following the indophenol blue colorimetric method at 630 nm. For NO_3_^−^-N, KCl extract was reduced through a copperized cadmium column, and the NO_3_^−^-N content was determined using the diazotization-coupling colorimetric method at 543 nm. The available nitrogen (AN) content was determined using the alkaline hydrolysis diffusion method (Zhang et al., 2022). The soil available potassium (AK) and available phosphorus (AP) contents were determined using the ammonium acetate extraction–flame photometry method and the sodium bicarbonate extraction–molybdenum–sulfuric acid spectrophotometry method, respectively (Miranda et al., 2001).

### 2.4 Extraction of genomic DNA from soil samples, PCR amplification, and high-throughput sequencing

#### 2.4.1 Sample DNA extraction

Total genomic DNA was extracted from the microbial community using a HiPure Soil DNA Kit (Magen, China, Catalog No. D3142) according to the manufacturer’s instructions. The integrity of the extracted DNA was assessed using 1% agarose gel electrophoresis, and the DNA concentration was determined using a Qubit 3.0 fluorometer (Thermo Fisher Scientific, United States).

#### 2.4.2 PCR amplification and sequencing library construction

Using the extracted DNA as a template, PCR amplification was performed to target the V3–V4 region of the 16S rRNA gene with barcoded primers 341F (5′-CCTACGGGNGGCWGCAG-3′) and 806R (5′-GGACTACHVGGGTATCTAAT-3′), and to target the ITS2 region with primers ITS3_KYO2 (5′-GATGAAGAACGYAGYRAA-3′) and ITS4 (5′-TCCTCCGCTTATTGATATGC-3′).

The PCR reaction mixture (50 μL) contained 10 μL of 5 × Q5^®^ Reaction Buffer, 10 μL of 5 × Q5^®^ High GC Enhancer, 1.5 μL of 2.5 mM dNTPs, 1.5 μL forward primer (10 μM), 1.5 μL reverse primer (10 μM), 0.2 μL Q5^®^ High-Fidelity DNA Polymerase, and 50 ng of template DNA (PCR-related reagents were purchased from New England Biolabs, United States). The PCR amplification program was run as follows: initial denaturation at 95 °C for 5 min; 30 cycles of denaturation at 95 °C for 1 min, annealing at 60 °C for 1 min, and extension at 72 °C for 1 min; and stable extension at 72 °C for 7 min. For every PCR reaction, a negative control (PCR negative control) comprising nuclease-free water instead of the template was included to monitor reagent contamination. The PCR products were examined using 2% agarose gel electrophoresis (the negative control showed no bands), purified using AMPure XP Beads (Beckman, CA, United States), and quantified using Qubit 3.0. Sequencing libraries were constructed using an Illumina DNA Prep Kit (Illumina, CA, United States) and assessed using the ABI StepOnePlus Real-Time PCR System (Thermo Fisher Scientific, United States). The qualified library was sequenced in PE250 mode on the Illumina NovaSeq 6000 platform.

#### 2.4.3 High-throughput sequencing data analysis

Quality control of the paired-end raw sequencing reads was performed using FASTP software (version 0.18.0), and the following reads were removed: (1) reads containing an uncertain nucleotide (N) proportion of ≥10%; (2) reads containing adapter sequences; and (3) low-quality reads (in which the proportion of bases with a Phred quality score ≤ 20 exceeded 50%). The resulting clean reads were used for subsequent analysis. Detailed sequencing statistics, including raw reads, clean reads, and effective reads, for every sample are provided in Supplementary Table S1. After quality control, paired-end reads were assembled into Tag sequences using FLASH software (version 1.2.11). with a minimum overlap length of 10 bp and a maximum mismatch ratio of 2%. The raw Tags were filtered as follows to yield high-quality clean Tags: truncation was performed at the first position in which three consecutive low-quality bases (quality value ≤ 3) occurred, and Tags for which the length of consecutive high-quality bases after truncation was less than 75% of the original Tag length were discarded.

#### 2.4.4 Data availability

The original sequencing data of this study has been deposited in the NCBI Sequence Read Archive (SRA), under the BioProject identifier PRJNA1477959. The data can be accessed at https://www.ncbi.nlm.nih.gov/sra/?term=PRJNA1477959.

### 2.5 Data processing and statistical analysis

To account for site effects, we used a linear mixed model (LMM) to analyze alpha diversity indices, soil properties, and enzyme activities, with group (healthy vs. diseased) as a fixed effect and sampling site as a random effect. This approach removes background variation among sites. To obtain absolute baseline values (presented as mean ± standard deviation), we performed independent-samples *t*-tests on alpha diversity indices, soil properties, soil enzyme activities, the relative abundances of the top ten microbial taxa at different taxonomic levels, and the relative abundances of indicator species. Levene’s test for equality of variances was conducted prior to each *t*-test. When variances were equal (*p* ≥ 0.05), we applied the independent-samples *t*-test assuming equal variances. When variances were unequal (*p* < 0.05), we applied Welch’s *t*-test, which does not assume equal variances. For beta diversity, we calculated the weighted and unweighted UniFrac distance matrices from operational taxonomic unit (OTU) representative sequences using the GuniFrac package in R. From the abundance table, we computed the Bray–Curtis distance matrix using the vegan package (version 2.5.3). We performed principal component analysis (PCA) and permutational analysis of variance (PERMANOVA) to test for overall differences in the microbial community structure between the two groups. We visualized taxonomic abundance statistics for every sample using the Krona package (version 2.6) in R to analyze the species composition. We created river plots of species abundance with ggplot2 (version 3.4.2) and used VennDiagram (version 1.7.3) to identify shared and unique species between groups. Using the set of differentially abundant species identified by Wilcoxon rank-sum tests at different taxonomic levels, we performed random forest analysis using the randomForest package (version 4.7.1.1) in R. This identified key indicator species distinguishing the healthy group from the diseased group. For functional prediction, we used PICRUSt2 (version 2.6.3) with the Kyoto Encyclopedia of Genes and Genomes (KEGG) database to predict bacterial community functions and the FUNGuild database (version 1.0) to predict fungal nutritional modes. To examine relationships between microbial communities (e.g., alpha diversity and abundance of dominant phyla and genera) and environmental factors (soil properties and enzyme activities), we used Spearman’s rank correlation (a nonparametric test) and generated plots using OriginPro 2025 (64-bit, version 10.2.0.188). We also calculated Pearson correlations among environmental factors using the psych package (version 1.8.4) in R. A Mantel test was performed using the vegan package (version 2.5.3) to assess links between the species composition and environmental factors. We used the ggcor package (version 0.9.6.1) in R to create combined heatmap–network diagrams, comparing how 16S and ITS data relate to environmental factors.

## 3 Results

### 3.1 Soil physicochemical properties and enzyme activities

**Figure 1:**
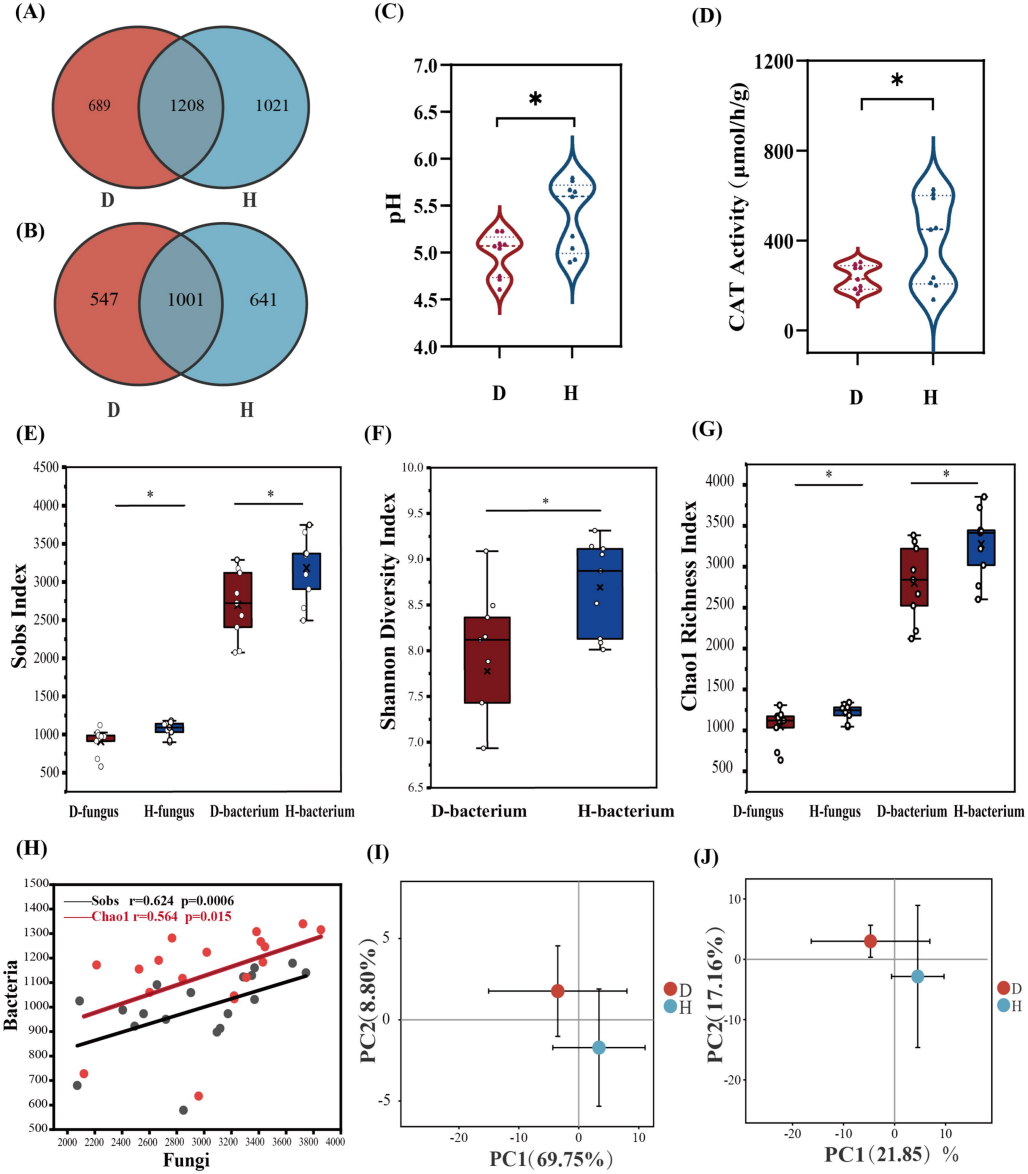
Analysis of microbial communities, soil physicochemical properties, and enzyme activities in the rhizosphere soil of healthy (H) and diseased tobacco plants (D). **(A)** Venn diagram showing the distribution of bacterial operational taxonomic unit (OTU) numbers; **(B)** Venn diagram showing the distribution of fungal OTU numbers; **(C)** Soil pH; **(D)** Catalase activity; **(E)** Box-and-whisker plot showing differences in the Sobs index of fungal and bacterial communities; **(F)** Box-and-whisker plot showing differences in the Shannon index of bacterial communities; **(G)** Box-and-whisker plot showing differences in the Chao1 index of bacterial and fungal communities; **(H)** Scatterplot showing the correlation between observed fungal and bacterial Sobs indices as well as the correlation between fungal and bacterial Chao1 indices (r = Spearman correlation coefficient; p = significance level); **(I)** Principal component analysis (PCA) based on bacterial community data; **(J)** PCA based on fungal community data. Statistically significant differences are indicated by asterisks (linear mixed model analysis): *p < 0.05 (*n* = 9).

Independent-samples *t*-tests and linear mixed model (LMM) analysis revealed significant differences in soil physicochemical properties and enzyme activities between healthy and diseased rhizosphere soils (Figures 1C,D; Supplementary Figure S1; Supplementary Tables S2, S5, S6). The pH and SOM, NO_3_^−^, AK, AP, and AN contents were higher in the rhizosphere soil of healthy tobacco plants than those in the rhizosphere soil of diseased tobacco plants (Figures 1C,D; Supplementary Figure S1; Supplementary Tables S2, S5). Among these, the pH reached a statistically significant difference (H: 5.39 ± 0.37; D: 4.98 ± 0.23) (Figure 1C; Supplementary Tables S2, S5). In contrast, the NH_4_^+^ content in healthy rhizosphere soil was lower than that in diseased rhizosphere soil (Supplementary Figure S1; Supplementary Tables S2, S3). The catalase (CAT), acid phosphatase (ACP), sucrase (SC), and urease (UE) activities in the rhizosphere soil of healthy tobacco plants were higher than those in diseased rhizosphere soil, with the CAT activity showing a statistically significant difference (H: 391.93 ± 196.05; D: 235.85 ± 55.30) (Figure 1D; Supplementary Tables S2, S6).

### 3.2 Microbial diversity analysis

Based on analysis of the OTU counts, there were significant differences in the microbial community composition of the rhizosphere soil between diseased and healthy tobacco plants. The number of unique bacterial OTUs in the rhizosphere soil of diseased and healthy plants was 689 and 1,021, respectively (Figure 1A), and the number of unique fungal OTUs was 547 and 641, respectively (Figure 1B). Alpha diversity analysis using a linear mixed model showed that the microbial diversity was significantly higher in the healthy group than in the diseased group (Figures 1E–G; Supplementary Tables S3, S7). Specifically, the Sobs and Chao1 indices for both bacteria and fungi and the Shannon index for bacteria were significantly elevated in healthy soils (Figures 1E–G; Supplementary Tables S3, S7). Additionally, there was a significant positive correlation between bacterial and fungal Sobs and Chao1 indices, suggesting a synergistic mechanism between bacterial and fungal communities in influencing soil-borne diseases (Figure 1H). We performed separate PERMANOVA analyses for the three locations to examine community differences between healthy and diseased soils at every site (Figure 1; Supplementary Figure S3). None of the individual site analyses reached statistical significance: FH (*R*^2^ = 0.301, *p* = 0.200), PL (*R*^2^ = 0.789, *p* = 0.100), and SM (*p* > 0.05). In contrast, an overall analysis combining all three sites showed a significant difference in the community structure between healthy and diseased soils (*R*^2^ = 0.086, *p* = 0.014), indicating that the disease effect was apparent after accounting for site-to-site variation (Figures 1I,J).

### 3.3 Microbial community composition

**Figure 2:**
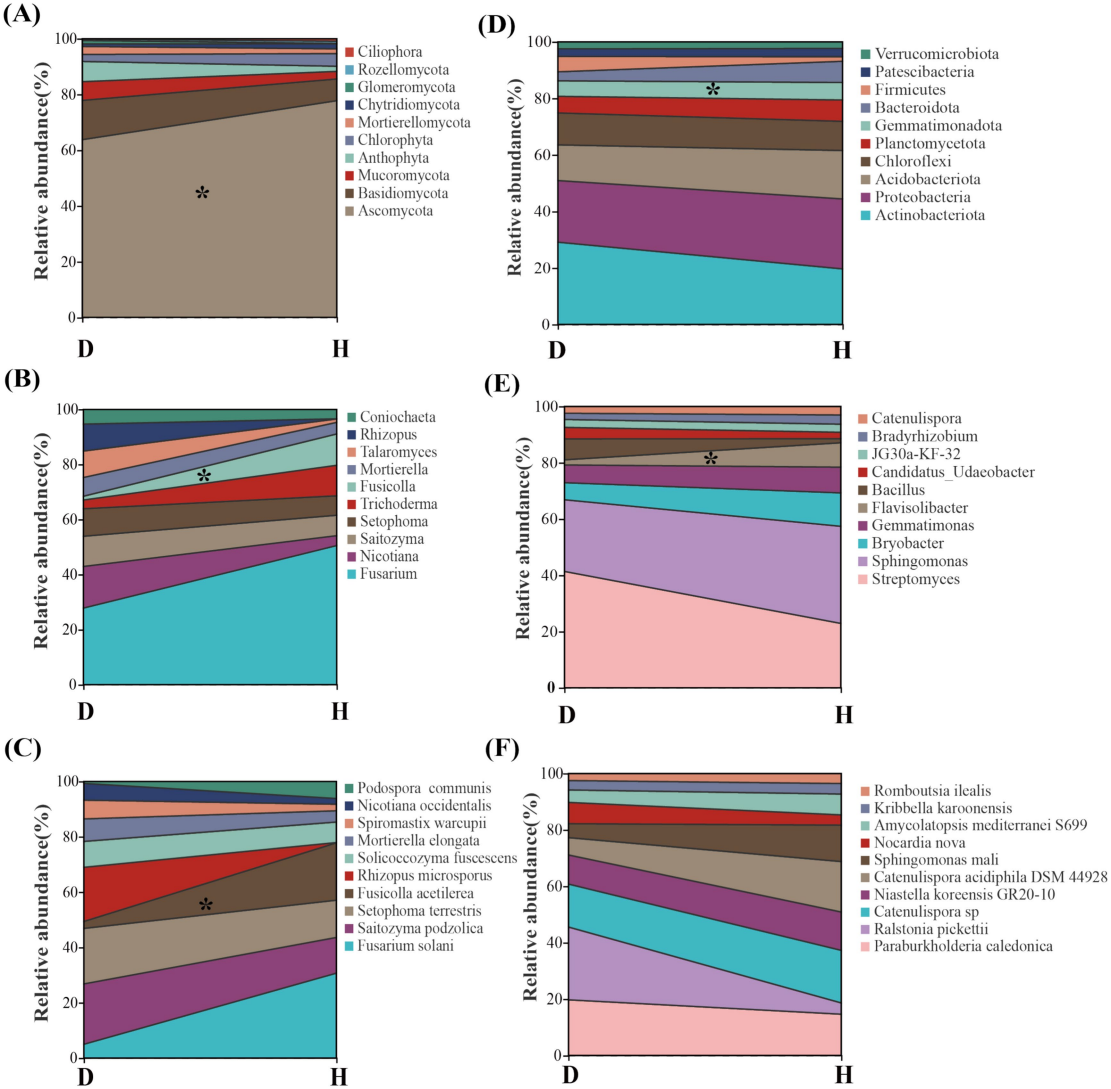
Microbial species composition in the rhizosphere soil of healthy (H) and diseased (D) tobacco plants. **(A)** Fungal phylum-level composition; **(B)** Fungal genus-level composition; **(C)** Fungal species-level composition; **(D)** Bacterial phylum-level composition; **(E)** Bacterial genus-level composition; **(F)** Bacterial species-level composition. Statistically significant differences are indicated by asterisks (Independent-samples *t*-tests): *p < 0.05 (*n* = 9).

**Figure 3:**
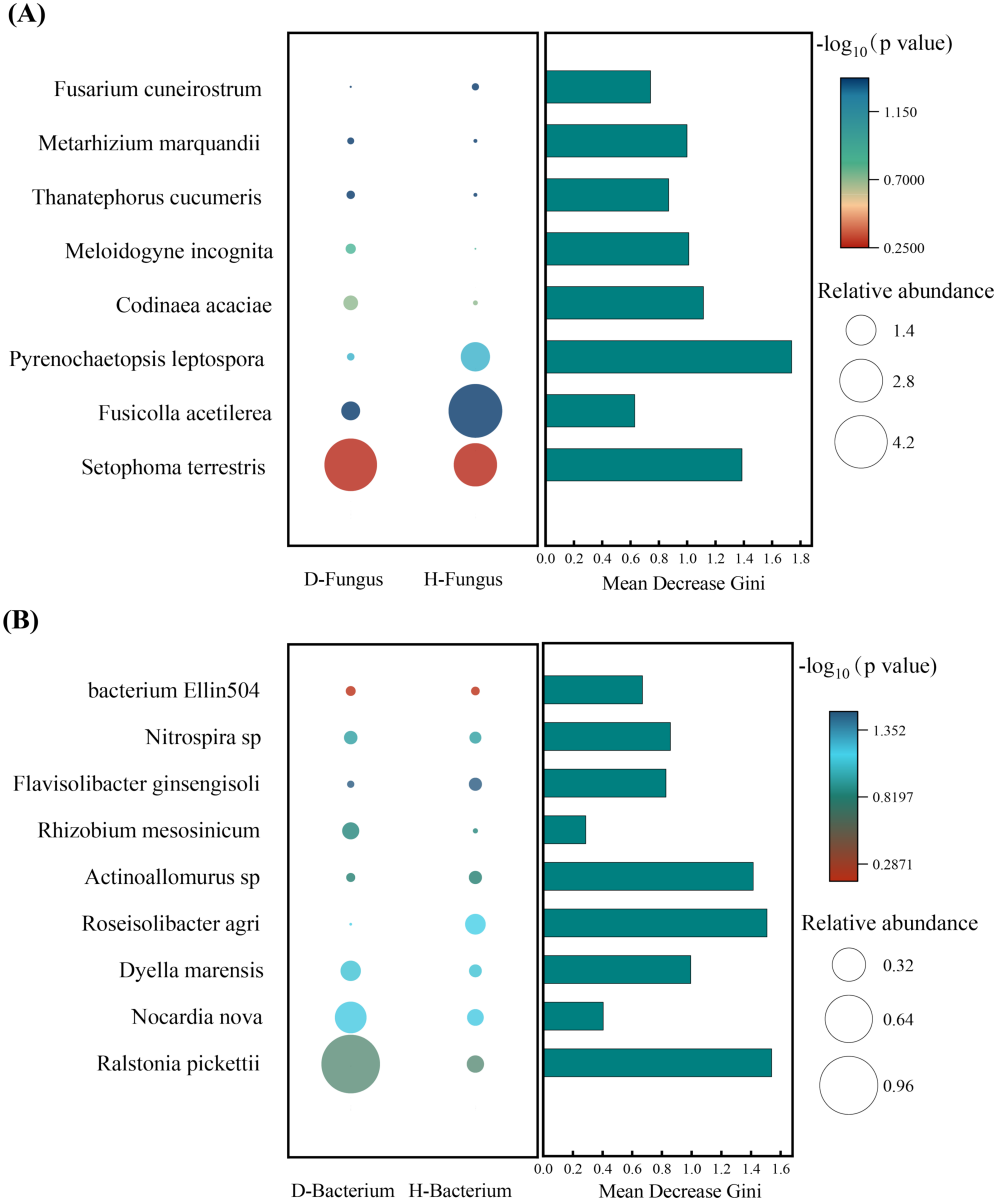
Random forest analysis identifying indicator species in the rhizosphere soil of healthy (H) and diseased (D) tobacco plants. **(A)** Fungal taxa: mean decrease in Gini values (importance ranking) are shown on the right, and –log_10_(*p*-values) on the left; the circle size represents the average relative abundance. **(B)** Bacterial taxa: same as in **(A)** (*n* = 9).

By analyzing the top 10 most abundant microbial taxa at the bacterial and fungal phylum, genus, and species levels, we found that the abundances of key microbial groups changed significantly in the rhizosphere soil of diseased tobacco plants. For fungi, the relative abundances of the species *Fusicolla acetilerea* (D: 0.52 ± 0.50; H: 4.28 ± 4.19), its genus *Fusicolla* (H: 4.47 ± 4.36; D: 0.57 ± 0.50), and the phylum Ascomycota (H: 75.92 ± 2.91; D: 62.23 ± 2.91) were significantly reduced by 87.96, 84.53, and 17.24%, respectively, in the rhizosphere soil of diseased plants compared to healthy soil, these differences were statistically significant (Figures 2A–C; Supplementary Tables S11–S13). For bacteria, the relative abundances of the genus *Flavisolibacter* (H: 2.30 ± 1.52; D: 0.61 ± 0.76) and phylum Bacteroidota (H: 6.70 ± 4.07; D: 2.89 ± 1.66) were significantly reduced by 79.07 and 57.0%, respectively, in the rhizosphere soil of diseased plants compared to healthy soil (Figures 3D,E; Supplementary Tables S8–10). Correlation analysis revealed that the aforementioned differences in microorganisms were closely related to each other. *Fusicolla_acetilerea*, *Fusicolla*, and Bacteroidota showed a significant, strong positive correlation and a highly significant positive correlation with *Flavisolibacter* (Supplementary Table S4).

### 3.4 Indicator species analysis

We performed random forest analysis at both the fungal and bacterial species levels to identify the key contributing species distinguishing the microbial community structures of healthy and diseased soils (Figure 3; Supplementary Figure S3). For fungi, the main species driving differences in the community structure between healthy and diseased soils were identified as *Setophoma terrestris*, *Fusicolla acetilerea*, *Pyrenochaetopsis leptospora*, *Codinaea acaciae*, *Meloidogyne incognita*, *Thanatephorus cucumeris*, *Metarhizium marquandii*, and *Fusarium cuneirostrum*. Among these, the average relative abundances of saprophytic fungi *Metarhizium marquandii* (D: 0.07 ± 0.05; H: 0.02 ± 0.02) and *Thanatephorus cucumeri*s (D: 0.10 ± 0.10; H: 0.02 ± 0.01) were significantly lower in healthy soil than in diseased soil, whereas the average relative abundances of *Fusicolla acetilerea* (D: 0.52 ± 0.50; H: 4.28 ± 4.19) and *Fusarium cuneirostrum* (D: 0.01 ± 0.01; H: 0.08 ± 0.09) were significantly higher in healthy soil than in diseased soil. *Fusicolla acetilerea* was the dominant fungal species in healthy soil (Figure 3A; Supplementary Table S14).

For bacteria, the main species contributing to differences in the bacterial community structure were identified as *Ralstonia pickettii*, *Nocardia nova*, *Dyella marensis*, *Roseisolibacter agri*, *Actinoallomurus* sp., *Rhizobium mesosinicum*, *Flavisolibacter ginsengisoli*, *Nitrospira* sp., and *bacterium Ellin504*. Among these, the average relative abundance of *Flavisolibacter ginsengisoli* (D: 0.01 ± 0.02; H: 0.05 ± 0.04) was significantly higher in healthy soil than in diseased soil, and *Flavisolibacter* was the dominant bacterial genus identified in healthy soil (Figure 3B; Supplementary Table S15).

### 3.5 Environmental factor correlation analysis

**Figure 4:**
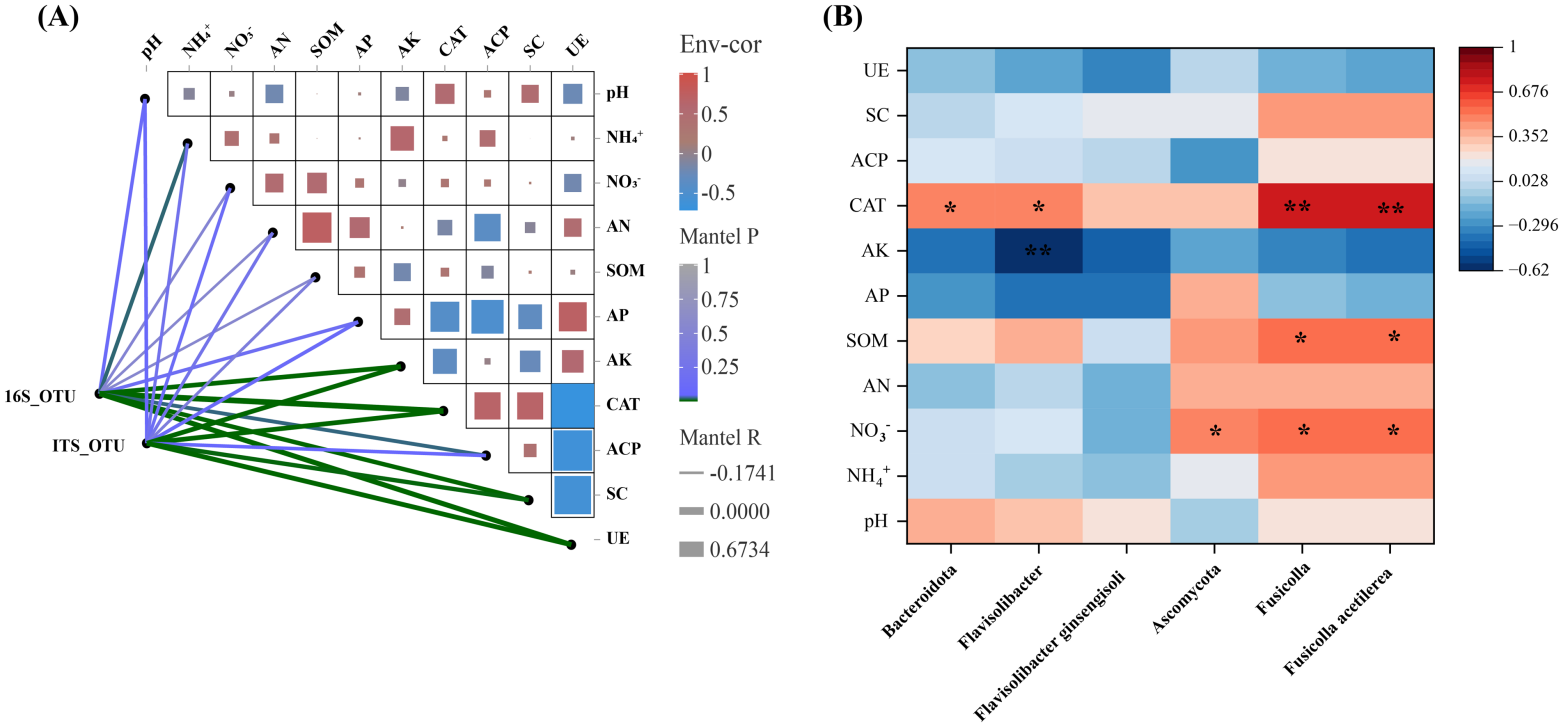
Correlation analysis between the soil microbial community structure and environmental factors. **(A)** Heatmap of Spearman correlation coefficients between environmental factors and 16S rRNA OTUs and ITS OTUs and Mantel test results. The degree of correlation is indicated by the line thickness (thicker lines represent stronger correlations). Statistical significance is represented by the line color: green lines indicate *p* < 0.05, and purple lines indicate no significant difference. The statistical significance of environmental factors is indicated by the square size (larger squares correspond to smaller *p*-values). Correlations between environmental factors are shown by the square color: red indicates a positive correlation (*r* > 0), and blue indicates a negative correlation (*r* < 0). **(B)** Spearman correlation heatmap of core microbial groups and environmental factors. Red indicates a positive correlation (*r* > 0), blue indicates a negative correlation (*r* < 0), and darker colors indicate stronger correlations. Significance levels: *p < 0.05; **p < 0.01 (*n* = 9).

Correlation analysis of environmental factors revealed that the OTUs of fungal and bacterial communities were significantly positively correlated with AK, CAT, SC, and UE (Figure 4A). Analysis of the characteristic microbial groups in healthy soil showed that the dominant bacteria, including *Flavisolibacter* and *Fusicolla acetilerea*, and fungi, including *Fusicolla* and *Flavisolibacter ginsengisoli*, were positively correlated with CAT and pH. *Flavisolibacter* exhibited a highly significant negative correlation with AK. Dominant fungal species *Fusicolla acetilerea*, its genus *Fusicolla*, and its phylum Ascomycota showed a significant positive correlation with the NO_3_^−^ content (Figure 4B). Further, *Fusicolla acetilerea* and *Fusicolla* were significantly positively correlated with the SOM content (Figure 4B).

### 3.6 Microbial function prediction analysis

**Figure 5:**
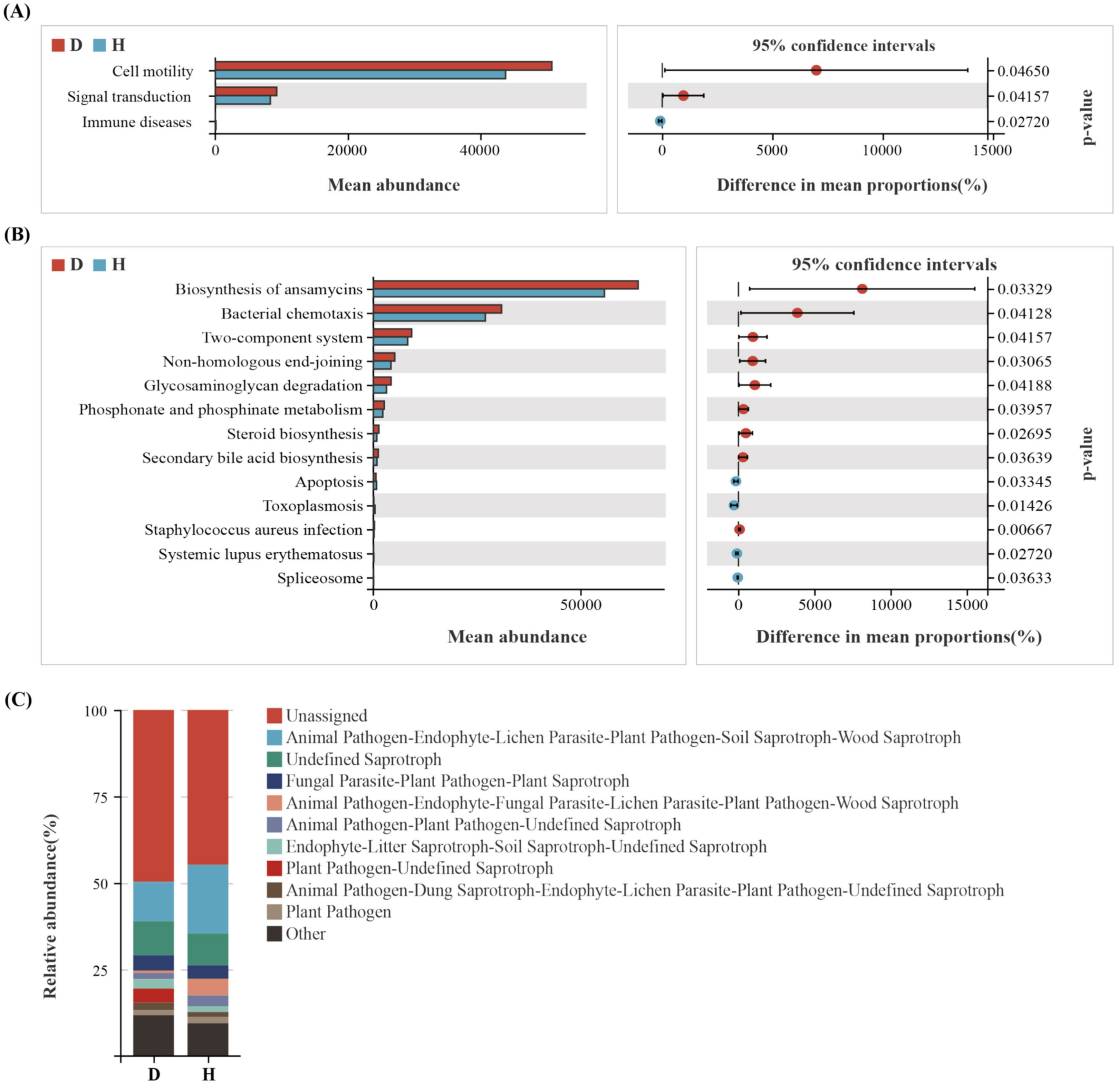
Prediction of microbial functions in the rhizosphere soil of healthy (H) and diseased (D) tobacco plants. **(A,B)** Welch’s test results for bacterial functional abundance at levels 2 and 3, respectively. For every panel, the left plot shows the mean abundance, and the right plot shows the *p*-value (*p* < 0.05). **(C)** FUNGuild prediction of fungal functional groups shown as a stacked bar chart (*n* = 9).

We performed predictive functional profiling using PICRUSt2 for bacterial communities (based on 16S rRNA gene data) and FUNGuild for fungal communities in order to determine the potential functional capabilities of rhizosphere microbial communities. These computational approaches infer the putative functions from taxonomic profiles and are widely used to generate hypotheses about microbial community functions in rhizosphere microbiome studies. Differential analysis of the bacterial functional abundance at KEGG pathway levels 2 and 3 was performed using Welch’s *t*-test. At level 2, the relative abundance of pathways related to cell motility and signal transduction was significantly lower in healthy soils than in diseased soils, but pathways associated with immune diseases showed the opposite pattern (Figure 5A). At the more detailed level 3, diseased soils exhibited a significantly higher relative abundance of pathways involved in metabolism, environmental adaptation, and stress responses. Specifically, pathways including biosynthesis of ansamycins, bacterial chemotaxis, two-component system, non-homologous end-joining, glycosylglycan degradation, phosphonate and phosphinate metabolism, steroid biosynthesis, and secondary bile acid biosynthesis were more abundant in diseased soils than in healthy soils (Figure 5B).

## 4 Discussion

Soil physicochemical properties and enzyme activities are key factors that influence plant health (Chu et al., 2021; Wang et al., 2022). Soil acidification accelerates the reproduction rate of RKNs and affects soil enzyme activities (Wang et al., 2009; Acosta-Martinez and Tabatabai, 2000; Yu et al., 2022). A decrease in the CAT activity leads to excessive hydrogen peroxide accumulation in the soil, damaging plant roots, weakening their nutrient absorption and disease resistance, and aggravating the disease (Wang et al., 2023). The pH and CAT activity in the rhizosphere soil of diseased tobacco plants were significantly lower than those in healthy plants, and the CAT activity decreased with decreasing soil pH. This observation suggests that a negative feedback process involving both decreased pH and reduced CAT activity occurs in diseased soils.

Soil microbial diversity is often used as a soil health indicator. A reduction in microbial diversity can lead to the occurrence of soil-borne diseases, whereas an increase can promote plant growth and suppress the disease incidence (Saleem et al., 2019). In this study, the diversity of bacteria and fungi in the rhizosphere soil of diseased tobacco plants was significantly reduced. There were significant positive correlations between bacterial and fungal diversity, as measured by both the Sobs and Chao1 indices. The concurrent decline in these indices in diseased soils may reflect reduced resource availability following root damage, compression of micro-niches, and environmental filtering effects triggered by the release of plant defense compounds. Together, these factors drive microbial communities toward lower diversity (Wang et al., 2025).

We found significant differences in the composition and predicted functional structure of microbial communities in the rhizosphere soil of diseased plants compared to healthy plants. Diseased soils were predicted to contain more microorganisms associated with the stress response, environmental adaptation, and stress tolerance. These differences reflect adaptive adjustments of the microbial community to changes in the soil environment, which could maintain community stability and support plant health under adverse conditions (Wang et al., 2025). *Fusicolla acetilerea*, which served both as an indicator species and a dominant fungal species, was significantly enriched in healthy soils. In healthy soil, the bacterial genus *Flavisolibacter* was the dominant bacterial genus, and *Flavisolibacter ginsengisoli* was an indicator species. Although little is currently known about *Fusicolla acetilerea* and *Flavisolibacter ginsengisoli* and no clear relationship with soil-borne diseases has been established, the genera Fusicolla and Flavisolibacter, which contain the dominant species identified above, were enriched in healthy soils as dominant fungal and bacterial genera, respectively. *Flavisolibacter* species are recognized as common plant growth-promoting bacteria (Kumar et al., 2023). Previous studies have shown that *Fusicolla* plays an important role in alleviating environmental stress on plant growth, controlling disease, and promoting bioremediation (Dong et al., 2023). Based on these findings, the enrichment of *Fusicolla acetilerea* and *Flavisolibacter ginsengisoli* in healthy soils suggests that they are potential biocandidate strains for disease control. In future studies, we plan to isolate and culture these strains and verify their functions using nematode quantification and pot inoculation experiments.

The interactions among soil physicochemical properties, enzyme activities, and soil microorganisms influence the occurrence and spread of soil-borne diseases (Ge et al., 2023). The results of this study indicate that CAT, SC, UE, and AK are the main environmental factors affecting the soil microbial community structure. Further, the key microbial groups identified in this study, namely, bacterial genus *Flavisolibacter*, bacterial species *Flavisolibacter ginsengisoli*, fungal genus *Fusicolla*, and fungal species *Fusicolla acetilerea*, showed positive correlations with the CAT activity and soil pH. *Flavisolibacte* and *Flavisolibacter ginsengisoli* are CAT-positive, meaning that they directly produce CAT (Baik et al., 2014). A higher soil nutrient content, pH, and soil oxidoreductase activity are more conducive to the enrichment and stable colonization of these health-indicating microorganisms; thus, the increased abundance of these microorganisms improves the soil nutrient status, enhances the biochemical activity, and maintains microecological health.

In summary, this study revealed systematic differences in soil properties, enzyme activities, and the microbial community structure and function between healthy and diseased tobacco rhizosphere soils. Based on these findings, we propose the following hypothesis: soil pH, CAT activity, and key nutrient-related properties are associated with the enrichment and colonization of health-associated microbial taxa, such as *Flavisolibacter* and *Fusicolla*, collectively maintaining a healthy soil microenvironment. In contrast, disease-related declines in the pH and enzyme activity may accompany a negative feedback process of soil degradation, loss of health-associated microbes, and exacerbated disease. These insights provide a theoretical basis and candidate microbial targets for controlling RKN disease through the targeted regulation of the soil microecology.

## 5 Conclusion

Multiple soil physicochemical, enzymatic, and microbial parameters differed systematically between the rhizosphere soils of healthy and RKN-infected tobacco plants. The pathological soil characteristics manifested as significant decreases in the pH, hydrogen peroxidase activity, microbial diversity, and nutrient content. In contrast, healthy soil was significantly enriched with key bacterial species *Flavisolibacter ginsengisoli* and key fungal species *Fusicolla acetilerea*, both of which were positively correlated with a higher CAT activity and NO_3_^−^-N and SOM contents. Bacterial community functions in diseased soil tended to be enriched in pathways related to metabolism, environmental adaptation, and stress responses.

In summary, this study revealed systematic differences in multiple physicochemical, enzymatic, and microbial indicators between healthy and diseased tobacco rhizosphere soils. Based on these findings, we propose a disease-related negative feedback hypothesis, in which declines in the soil pH and CAT activity may be accompanied by rhizosphere microenvironment degradation and a reduction in health-associated microbial taxa. Based on the above findings, effective control of root-knot nematodes necessitates the coordinated regulation of soil pH, enzyme activity, and microbial communities to preserve a healthy rhizosphere microecology.

## Data Availability

The original contributions presented in the study are included in the article/Supplementary material, further inquiries can be directed to the corresponding authors.
