# Supplementary material for: Rhizosphere microecological characteristics associated with tobacco root-knot nematode disease
Source: Front Microbiol. 2026 Jul 16;17:1874628. doi: 10.3389/fmicb.2026.1874628 (PMC13426051; doi:10.3389/fmicb.2026.1874628)
Supplement: Supplementary file 1 [file Data_Sheet_1.PDF]

SUPPLEMENTARY INFORMATION

Rhizosphere Microecological Characteristics Associated with Tobacco Root-Knot Nematode Disease

……

Tables: 15

Figures: 4

List of supporting information

Table

Table S1 Statistical summary of data preprocessing and quality control.

Table S2 Comparison of rhizosphere soil indicators between healthy and diseased tobacco plants based on a linear mixed model (LMM).

Table S3 Comparison of rhizosphere soil alpha diversity between healthy and diseased tobacco plants based on a linear mixed model (LMM).

Table S4 Spearman correlation analysis of bacterial and fungal taxonomic groups.

Table S5 Comparison of soil chemical properties between healthy (H) and diseased (D) tobacco rhizosphere soil

Table S6 Comparison of soil enzyme activities in the rhizosphere soil of healthy (H) and diseased (D) tobacco plants

Table S7 Comparison of alpha diversity indices in rhizosphere soil between healthy (H) and diseased (D) tobacco plants

Table S8 Comparison of bacterial phylum relative abundances in rhizosphere soil between healthy (H) and diseased (D) tobacco plants

Table S9 Comparison of bacterial genus relative abundances in rhizosphere soil between healthy (H) and diseased (D) tobacco plants

Table S10 Comparison of bacterial species relative abundances in rhizosphere soil between healthy (H) and diseased (D) tobacco plants

Table S11 Comparison of fungal phylum relative abundances in rhizosphere soil between healthy (H) and diseased (D) tobacco plants

Table S12 Comparison of top 10 fungal genera relative abundances in rhizosphere soil between healthy (H) and diseased (D) tobacco plants

Table S13 Comparison of top 10 fungal species relative abundances in rhizosphere soil between healthy (H) and diseased (D) tobacco plants

Table S14 Comparison of indicator fungal species relative abundances in rhizosphere soil between healthy (H) and diseased (D) tobacco plants

Table S15 Comparison of indicator bacterial species relative abundances in

rhizosphere soil between healthy (H) and diseased (D) tobacco plants

Figure

Figure S1. Analysis of soil physicochemical properties and enzyme activities in the rhizosphere soil of healthy (H) and diseased (D) tobacco plants.

Figure S2. Rarefaction curves based on Good's coverage for bacterial (A) and fungal (B) communities in the diseased (D) and healthy (H) groups

Figure S3. OTU-level PERMANOVA of rhizosphere soil microbial communities in healthy and diseased tobacco plants from different plots.

Figure S4. Random forest analysis identifying indicator species in the rhizosphere soil of healthy (H) and diseased (D) tobacco plants.

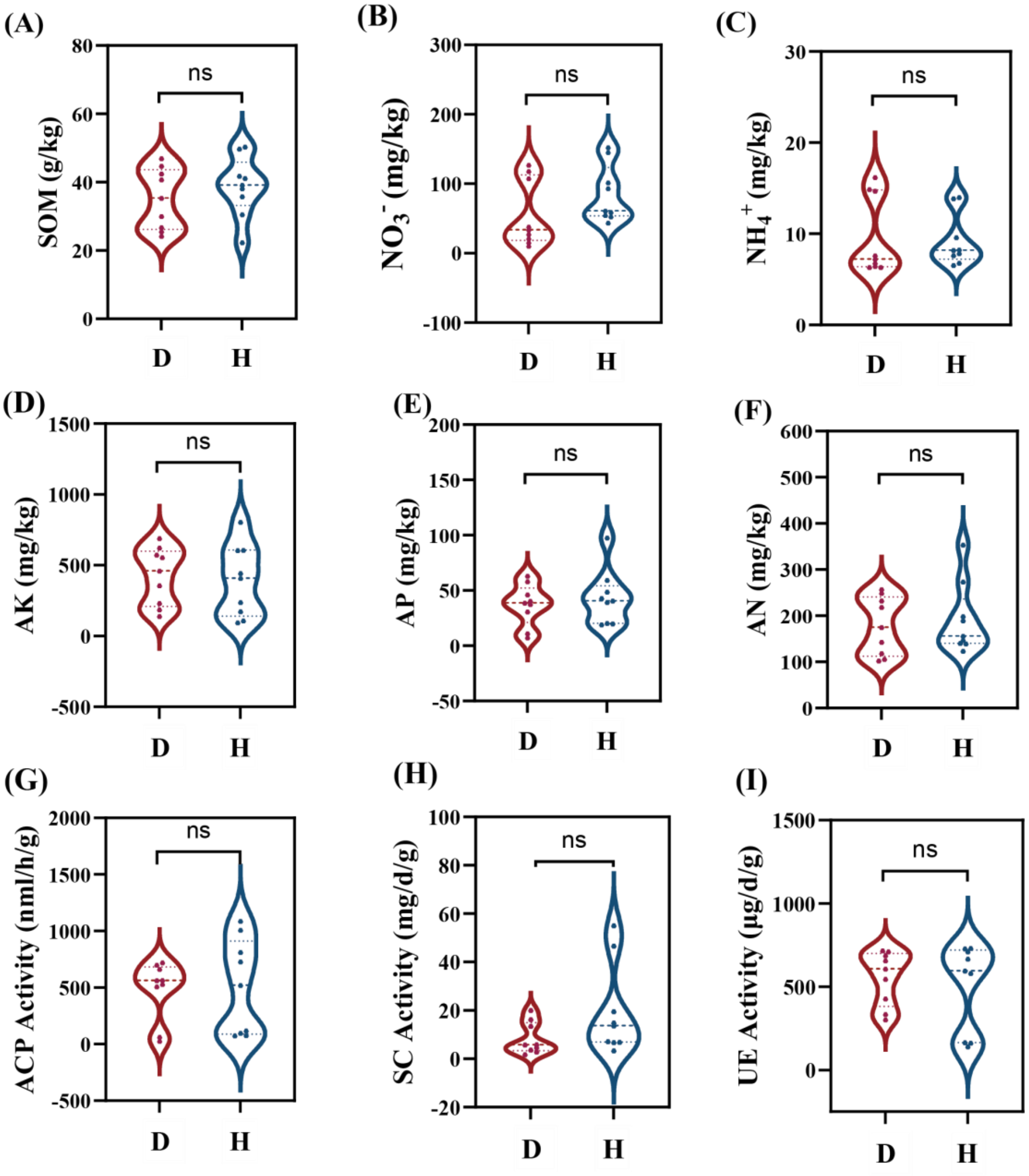

Figure S2. Analysis of soil physicochemical properties and enzyme activities in the rhizosphere soil of healthy (H) and diseased (D) tobacco plants. (A) Soil organic matter (SOM); (B) Nitrate nitrogen (NO_3^-); (C) Ammonium nitrogen (NH_4^+); (D) Available potassium (AK); (E) Available phosphorus (AP); (F) Available nitrogen (AN); (G) Acid phosphatase (ACP) activity; (H) Sucrase (SC) activity; (I) Urease (UE) activity. Statistically significant differences are indicated by asterisks (linear mixed model analysis): * $p < 0.05$; "ns" indicates not significant (n = 9).

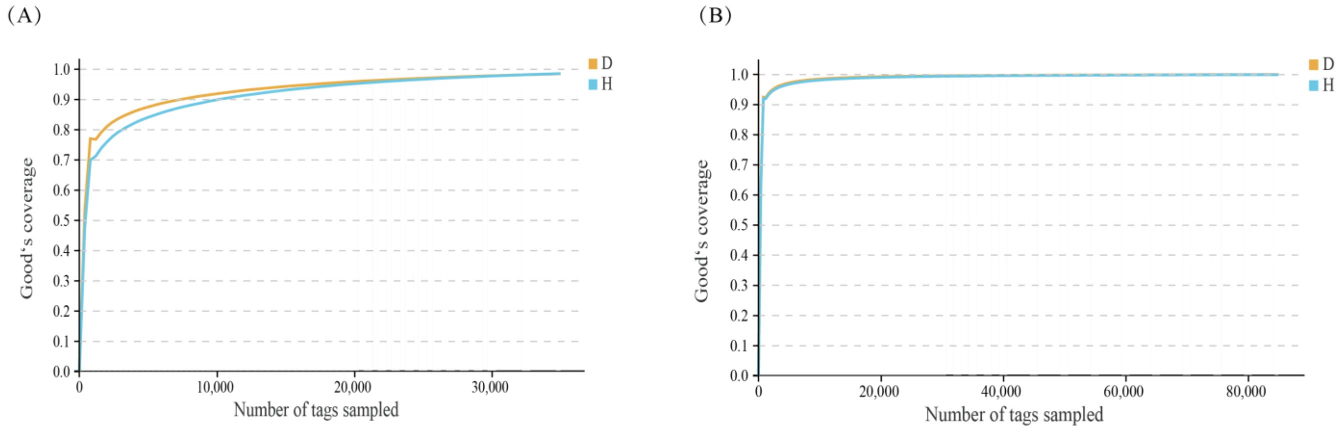

Figure S2. Rarefaction curves based on Good's coverage for bacterial (A) and fungal (B) communities in the diseased (D) and healthy (H) groups (n = 9).

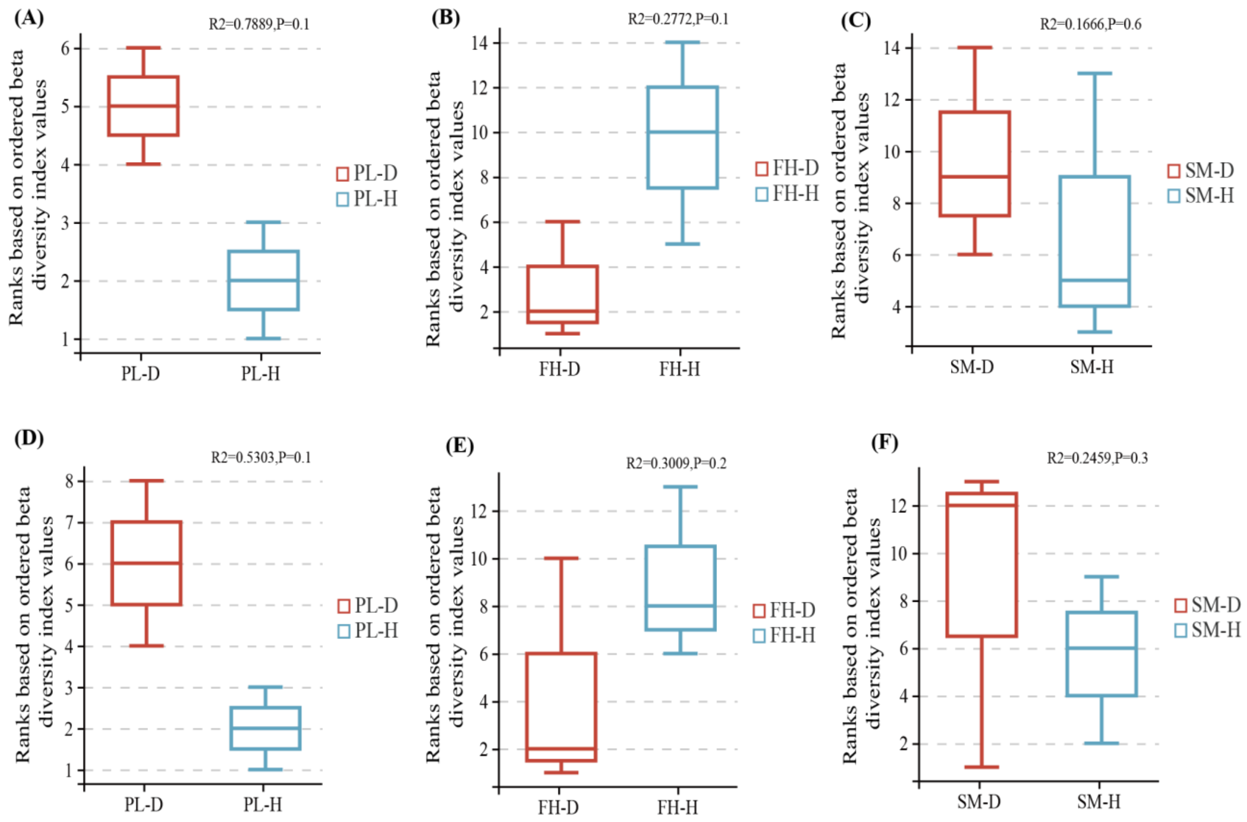

Figure S3. OTU-level PERMANOVA of rhizosphere soil microbial communities in healthy and diseased tobacco plants from different plots. Panels (A), (B), and (C) represent bacterial communities; panels (D), (E), and (F) represent fungal communities. PL, Fenghe Town, Xundian County, Kunming; SM, Aziying Township, Songming County, Kunming; D, diseased; H, healthy. Boxplots show the distribution of beta diversity rankings for each group. PERMANOVA (Adonis) results are shown above each panel. R^2 represents the proportion of variance in microbial community dissimilarity explained by the treatment (healthy vs. diseased), ranging from 0 to 1, with higher values indicating greater treatment effects; P represents the significance level from permutation testing ($p < 0.05$ indicates a statistically significant difference in the community structure between healthy and diseased groups) (n = 3).

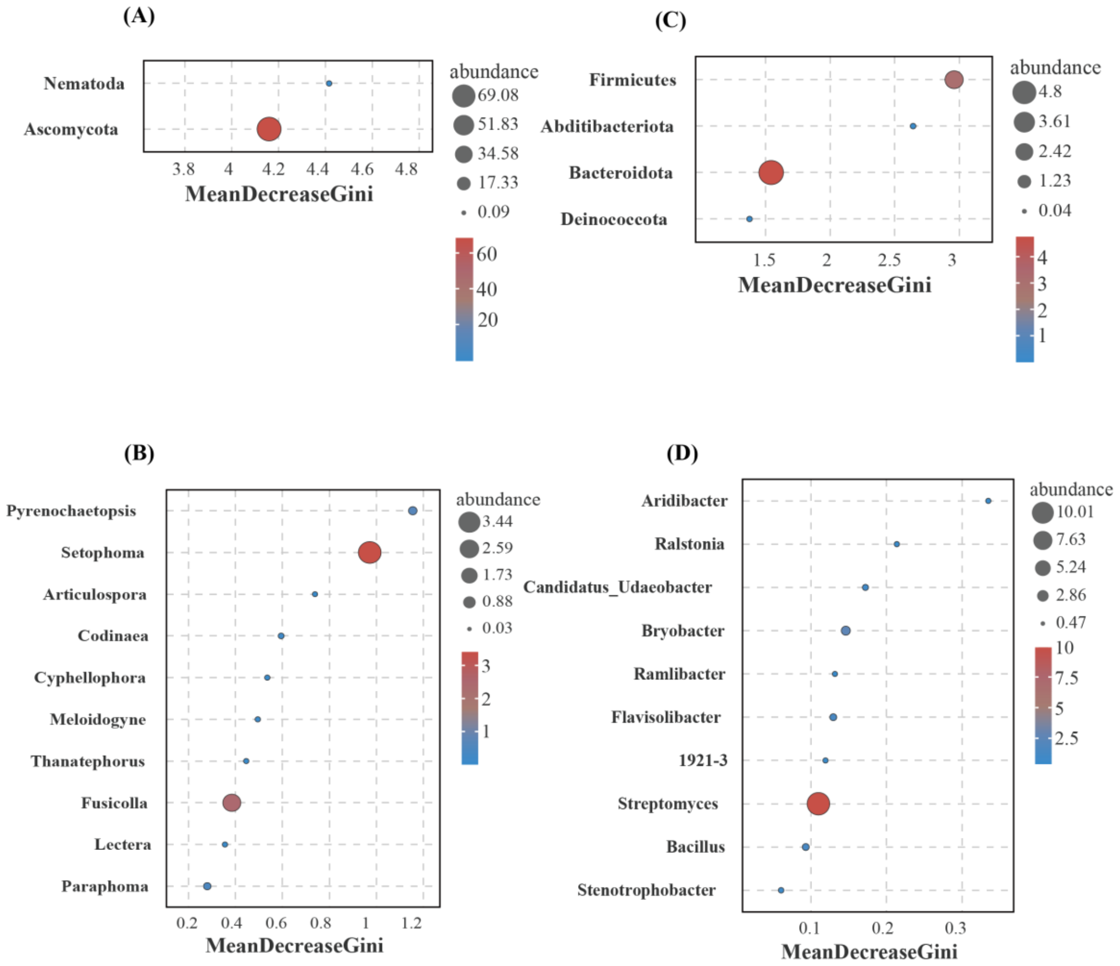

Figure S4. Random forest analysis identifying indicator species in the rhizosphere soil of healthy (H) and diseased (D) tobacco plants. (A) Fungal kingdom level; (B) Fungal genus level; (C) Bacterial kingdom level; (D) Bacterial genus level. The size of the circle and depth of the color indicate the magnitude of the abundance (n = 9).

Table S1 Statistical summary of data preprocessing and quality control.

	Sample Name	Raw Reads	Clean Reads	Raw Tags	Clean Tags	Chimera	Effective Tags	Effective Ratio (%)
	FH-D-1	126,741	126,525	124,608	123,825	30,228	93,597	73.85
	FH-D-2	128,611	128,415	126,619	125,895	37,054	88,841	69.08
	FH-D-3	135,681	135,471	133,430	132,690	35,463	97,227	71.66
	FH-H-1	125,509	125,328	123,345	122,624	36,815	85,809	68.37
D	FH-H-2	136,336	136,139	133,997	133,205	40,570	92,635	67.95
	FH-H-3	126,644	126,486	124,427	123,552	38,388	85,164	67.25
	PL-D-1	124,273	124,110	122,278	121,636	39,753	81,883	65.89
	PL-D-2	121,447	121,329	119,683	115,375	31,741	83,634	68.86
	PL-D-3	130,308	130,119	128,141	127,361	38,946	88,415	67.85
	PL-H-1	136,147	135,948	133,783	132,997	40,204	92,793	68.16
	PL-H-2	131,540	131,369	129,161	128,483	40,574	87,909	66.83
	PL-H-3	123,384	123,217	121,343	120,696	38,665	82,031	66.48
	SM-D-1	127,220	127,025	124,744	123,985	39,265	84,720	66.59
H	SM-D-2	128,700	128,508	126,377	125,665	40,654	85,011	66.05
	SM-D-3	128,301	128,117	125,657	124,860	39,756	85,104	66.33
	SM-H-1	136,011	135,844	133,595	132,769	43,921	88,848	65.32
	SM-H-2	136,321	136,098	134,078	133,234	41,080	92,154	67.60
	SM-H-3	134,837	134,617	132,427	131,591	41,005	90,586	67.18

For each sample, we recorded the following metrics: the number of raw paired-end reads (Raw PE); the number of high-quality paired-end reads after quality filtering (Clean PE); the number of tags initially obtained after merging overlapping reads (Raw Tags); the number of high-quality tags after quality filtering (Clean Tags); the number of chimeric tags detected during OTU clustering (Chimera); and the number of high-quality tags remaining after chimera removal, which were used as effective tags for downstream analysis (Effective Tags). We also calculated the effective ratio,

defined as the percentage of raw paired-end reads that remained effective tags (Effective Ratio, %).

Table S2 Comparison of rhizosphere soil indicators between healthy and diseased tobacco plants based on a linear mixed model (LMM).

Indicator	H	D	Estimate	p-value	ICC
**pH**	**5.394**	**4.983**	**-0.411**	**0.0092**	**0.067**
NH_4^+ (mg/kg)	9.207	9.621	0.414	0.6918	0.717
NO_3^- (mg/kg)	85.015	55.504	-29.511	0.1333	0.243
AN (mg/kg)	191.551	178.272	-13.279	0.6557	0.281
SOM (g/kg)	38.790	35.214	-3.577	0.402	0.000
AP (mg/kg)	43.269	37.157	-6.112	0.4763	0.429
AK (mg/kg)	387.894	424.661	36.767	0.4976	0.814
**CAT (cmol/kg)**	**391.928**	**235.853**	**-156.075**	**0.0164**	**0.248**
ACP (nmml/h/g)	503.798	482.886	-20.912	0.8925	0.209
SC (mg/d/g)	19.443	8.364	-11.079	0.1072	0.042
UE (μg/g/h)	499.043	555.568	56.525	0.4339	0.610

H, healthy group; D, diseased group; Estimate, estimated treatment effect (difference between diseased and healthy groups); p-value, significance level; ICC, intraclass correlation coefficient, indicating the proportion of total variance attributable to site differences. Significant p-values ($p < 0.05$) are indicated in bold ($n = 9$).

Table S3 Comparison of rhizosphere soil alpha diversity between healthy and diseased tobacco plants based on a linear mixed model (LMM).

Indicator		H	D	Estimate	p-value	ICC
16S	**sobs**	**3180.333**	**2696.667**	**-483.667**	**0.034**	**0.000**
	**shannon**	**8.691**	**7.775**	**-0.916**	**0.021**	**0.132**
	simpson	0.980	0.961	-0.020	0.142	0.272
	**chao**	**3274.381**	**2804.395**	**-469.986**	**0.038**	**0.000**
ITS	**sobs**	**1067.667**	**911.556**	**-156.111**	**0.015**	**0.235**
	shannon	5.746	5.747	0.001	0.998	0.141
	simpson	0.930	0.935	0.006	0.748	0.233
	**chao**	**1218.129**	**1051.327**	**-166.802**	**0.033**	**0.246**

H, healthy group; D, diseased group; Estimate, estimated treatment effect (difference between diseased and healthy groups); p-value, significance level; ICC, intraclass correlation coefficient, indicating the proportion of total variance attributable to site differences. Significant p-values ($p < 0.05$) are indicated in bold ($n = 9$).

Table S4 Spearman correlation analysis of bacterial and fungal taxonomic groups.

	Ascomycota	*Fusicolla*	*Fusicolla_acetilerea*
Bacteroidota	0.310	0.546*	0.546*
Flavisolibacter	0.360	0.703**	0.717**

*Indicates a significant correlation ($p < 0.05$), ** indicates an extremely significant correlation ($p < 0.01$) (n = 9).

Table S5 Comparison of soil chemical properties between healthy (H) and diseased (D) tobacco rhizosphere soil

Indicator	pH	NH_4^+ (mg/kg)	NO_3^- (mg/kg)	AN (mg/kg)
**D**	4.98 ± 0.23 b	9.62 ± 4.28 a	55.50 ± 47.65 a	178.27 ± 62.75 a
**H**	5.39 ± 0.37 a	9.21 ± 2.83 a	85.02 ± 40.94 a	191.55 ± 76.01 a

Indicator	AP (mg/kg)	AK (mg/kg)	SOM (g/kg)
**D**	37.16 ± 18.77 a	424.66 ± 202.99 a	35.21 ± 8.85 a
**H**	43.27 ± 24.81 a	387.89 ± 251.48 a	38.79 ± 8.78 a

Values are presented as mean ± standard deviation (SD). Different lowercase letters within the same column indicate significant differences between treatments ($p < 0.05$, t-test). D = Diseased, H = Healthy. NH_4^+ = ammonium nitrogen, NO_3^- = nitrate nitrogen, AN = alkaline hydrolysis nitrogen, AP = available phosphorus, AK = available potassium, SOM = soil organic matter. (n=9).

Table S6 Comparison of soil enzyme activities in the rhizosphere soil of healthy (H) and diseased (D) tobacco plants

Indicator	CAT (cmol/kg)	ACP (nmml/h/g)	SC (mg/d/g)	UE (μg/g/h)
**D**	235.85 ± 55.30 b	482.89 ± 259.06 a	8.36 ± 6.61 a	555.57 ± 162.09 a
**H**	391.93 ± 196.05 a	503.80 ± 421.21 a	19.44 ± 18.69 a	499.04 ± 261.84 a

Values are presented as mean ± standard deviation (SD). Different lowercase letters within the same column indicate significant differences between treatments ($p < 0.05$, t-test). D = Diseased, H = Healthy. CAT: Catalase； SC: Sucrase; ACP: Acid phosphatase; UE: Urease. (n=9).

Table S7 Comparison of alpha diversity indices in rhizosphere soil between healthy (H) and diseased (D) tobacco plants

Index		Sobs	Shannon	Simpson	Chao1
16S	D	2696.67 ± 452.88 b	7.78 ± 1.05 b	0.96 ± 0.04 a	2804.40 ± 461.43 b
	H	3180.33 ± 428.72 a	8.69 ± 0.51 a	0.98 ± 0.02 a	3274.38 ± 416.98 a
ITS	D	911.56 ± 171.99 b	5.75 ± 0.90 a	0.94 ± 0.04 a	1051.33 ± 222.60 a
	H	1067.67 ± 101.17 a	5.75 ± 0.81 a	0.93 ± 0.04 a	1218.13 ± 104.61 a

Values are presented as mean ± standard deviation (SD). Different lowercase letters within the same column indicate significant differences between treatments ($p < 0.05$, t-test). D = Diseased, H = Healthy. (n=9).

Table S8 Comparison of bacterial phylum relative abundances in rhizosphere soil between healthy (H) and diseased (D) tobacco plants

Phylum(%)	Proteobacteria	Acidobacteriota	Actinobacteriota	Chloroflexi	Planctomycetota
D	19.89 ± 8.48 a	11.51 ± 3.91 a	26.58 ± 14.03 a	10.31 ± 3.29 a	5.36 ± 2.41 a
H	22.10 ± 4.66 a	15.29 ± 4.93 a	17.57 ± 10.90 a	9.22 ± 3.82 a	6.74 ± 1.60 a

Phylum(%)	Gemmatimonadota	Bacteroidota	Firmicutes	Patescibacteria	Verrucomicrobiota
D	5.01 ± 1.63 a	2.89 ± 1.66 b	4.97 ± 5.37 a	2.45 ± 1.71 a	2.27 ± 1.62 a
H	5.52 ± 1.75 a	6.70 ± 4.07 a	1.43 ± 0.71 a	2.64 ± 1.04 a	2.28 ± 1.21 a

Values are presented as mean ± standard deviation (SD). Different lowercase letters within the same column indicate significant differences between treatments ($p < 0.05$, t-test). D = Diseased, H = Healthy. Only the top 10 phyla by average relative abundance are shown. Phylum names follow current taxonomic nomenclature. (n=9).

Table S9 Comparison of bacterial genus relative abundances in rhizosphere soil between healthy (H) and diseased (D) tobacco plants

Genus(%)	*Sphingomonas*	*Bryobacter*	*Streptomyces*	*Gemmatimonas*	*Flavisolibacter*
D	8.59 ± 4.06 a	2.04 ± 1.37 a	13.94 ± 13.42 a	2.12 ± 1.04 a	0.61 ± 0.76 b
H	9.17 ± 3.09 a	3.16 ± 1.15 a	6.08 ± 8.62 a	2.43 ± 1.22 a	2.30 ± 1.52 a

Genus(%)	*Bacillus*	*Candidatus_Udaeobacter*	*JG30a-KF-32*	*Bradyrhizobium*	*Catenulispora*
D	2.51 ± 4.20 a	1.38 ± 1.29 a	0.95 ± 0.50 a	0.75 ± 0.33 a	0.81 ± 0.37 a
H	0.39 ± 0.38 a	0.61 ± 0.91 a	0.76 ± 1.34 a	0.87 ± 0.49 a	0.80 ± 0.96 a

Values are presented as mean ± standard deviation (SD). Different lowercase letters within the same column indicate significant differences between treatments ($p < 0.05$, t-test). D = Diseased, H = Healthy. Only the top 10 genera by average relative abundance are shown. (n=9).

Table S10 Comparison of bacterial species relative abundances in rhizosphere soil between healthy (H) and diseased (D) tobacco plants

Species (%)	*Ralstonia_ pickettii*	*Catenulispora_ sp*	*Paraburkholderia _caledonica*	*Niastella_koreens is_GR20-10*	*Catenulispora_ acidiphila_DSM_ 44928*
D	0.96 ± 1.81 a	0.56 ± 0.33 a	0.73 ± 1.27 a	0.38 ± 0.44 a	0.23 ± 0.14 a
H	0.08 ± 0.17 a	0.39 ± 0.36 a	0.31 ± 0.49 a	0.29 ± 0.68 a	0.38 ± 0.66 a

Species (%)	*Sphingomonas_ mali*	*Nocardia_ nova*	*Amycolatopsis_ mediterranei_S6 99*	*Kribbella_ karoonensis*	*Romboutsia_ ilealis*
D	0.18 ± 0.09 a	0.28 ± 0.28 a	0.16 ± 0.13 a	0.12 ± 0.12 a	0.09 ± 0.06 a
H	0.27 ± 0.15 a	0.08 ± 0.07 a	0.16 ± 0.19 a	0.08 ± 0.08 a	0.08 ± 0.06 a

Values are presented as mean ± standard deviation (SD). Different lowercase letters within the same column indicate significant differences between treatments ($p < 0.05$, t-test). D = Diseased, H = Healthy. Only the top 10 species by average relative abundance are shown. (n=9).

Table S11 Comparison of fungal phylum relative abundances in rhizosphere soil between healthy (H) and diseased (D) tobacco plants

Phylum (%)	Basidiomycota	Mucoromycota	Ascomycota	Anthophyta	Chlorophyta
H	7.60 ± 1.73 a	2.65 ± 1.35 a	75.92 ± 2.91 a	1.80 ± 0.40 a	4.44 ± 1.76 a
D	13.73 ± 1.73 a	6.55 ± 1.35 a	62.23 ± 2.91 b	7.08 ± 0.40 a	2.45 ± 1.76 a

Phylum (%)	Mortierellomycota	Chytridiomycota	Glomeromycota	Rozellomycota	Ciliophora
H	1.66 ± 0.51 a	1.77 ± 0.67 a	0.13 ± 0.02 a	0.78 ± 0.63 a	0.87 ± 0.39 a
D	2.79 ± 0.51 a	0.91 ± 0.67 a	1.20 ± 0.02 a	0.51 ± 0.63 a	0.10 ± 0.39 a

Values are presented as mean ± standard deviation (SD). Different lowercase letters within the same column indicate significant differences between treatments ($p < 0.05$, t-test). D = Diseased, H = Healthy. Only the top 10 phyla by average relative abundance are shown. (n=9).

Table S12 Comparison of top 10 fungal genera relative abundances in rhizosphere soil between healthy (H) and diseased (D) tobacco plants

Genus (%)	*Nicotiana*	*Saitozyma*	*Fusarium*	*Setophoma*	*Trichoderma*
D	6.19 ± 8.04 a	4.50 ± 2.52 a	11.42 ± 7.11 a	4.10 ± 3.21 a	1.30 ± 1.47 a
H	1.41 ± 1.26 a	2.91 ± 2.49 a	19.87 ± 11.17 a	2.78 ± 5.46 a	4.36 ± 8.03 a

Genus (%)	*Fusicolla*	*Mortierella*	*Talaromyces*	*Rhizopus*	*Coniochaeta*
D	0.57 ± 0.50 a	2.79 ± 2.88 a	3.94 ± 7.75 a	4.01 ± 10.79 a	2.19 ± 1.37 a
H	4.47 ± 4.36 b	1.66 ± 1.54 a	0.48 ± 0.28 a	0.02 ± 0.02 a	1.34 ± 1.10 a

Values are presented as mean ± standard deviation (SD). Different lowercase letters within the same column indicate significant differences between treatments ($p < 0.05$, t-test). D = Diseased, H = Healthy. Only the top 10 genera by average relative abundance are shown. (n=9).

Table S13 Comparison of top 10 fungal species relative abundances in rhizosphere soil between healthy (H) and diseased (D) tobacco plants

Species (%)	Saitozyma_ podzolica	Setophoma_ terrestris	Fusarium_ solani	Fusicola_ acetilerea	Rhizopus_ microsporus
H	2.66 ± 2.65 a	2.78 ± 5.46 a	6.34 ± 9.05 a	4.28 ± 4.19 a	0.02 ± 0.02 a
D	4.45 ± 2.52 a	4.10 ± 3.21 a	1.02 ± 0.78 a	0.52 ± 0.50 b	4.00 ± 10.79 a

Species (%)	Solicoceozyma_ fuscescens	Mortierella_ elongata	Spiromastix_ warcupii	Nicotiana_ occidentalis	Podospora_ communis
H	1.53 ± 2.13 a	0.84 ± 1.06 a	0.49 ± 0.64 a	0.43 ± 0.44 a	1.28 ± 2.44 a
D	1.91 ± 1.27 a	1.66 ± 2.63 a	1.39 ± 2.10 a	1.24 ± 1.25 a	0.15 ± 0.17 a

Values are presented as mean ± standard deviation (SD). Different lowercase letters within the same column indicate significant differences between treatments ($p < 0.05$, t-test). D = Diseased, H = Healthy. Only the top 10 species by average relative abundance are shown. (n=9).

Table S14 Comparison of indicator fungal species relative abundances in rhizosphere soil between healthy (H) and diseased (D) tobacco plants

Species (%)	*Fusicolla_ acetilerea*	*Pyrenochaetopsis_ leptospora*	*Setophoma_ terrestris*	*Codinaea_ acaciae*
D	0.52 ± 0.50 b	0.08 ± 0.06 a	4.10 ± 3.21 a	0.32 ± 0.61 a
H	4.28 ± 4.19 a	1.26 ± 1.67 a	2.78 ± 5.46 a	0.03 ± 0.03 a

Species (%)	*Meloidogyne_ incognita*	*Thanatephorus_ cucumeris*	*Metarhizium_ marquandii*	*Fusarium_ cuneirostrum*
D	0.15 ± 0.26 a	0.10 ± 0.10 a	0.07 ± 0.05 a	0.01 ± 0.01 b
H	0.01 ± 0.01 a	0.02 ± 0.01 b	0.02 ± 0.02 a	0.08 ± 0.09 a

Values are presented as mean ± standard deviation (SD). Different lowercase letters within the same column indicate significant differences between treatments ($p < 0.05$, t-test). D = Diseased, H = Healthy. Species listed are those identified as key contributors by random forest analysis. (n=9).

Table S15 Comparison of indicator bacterial species relative abundances in rhizosphere soil between healthy (H) and diseased (D) tobacco plants

Species (%)	*Rhizobium_ mesosinicum*	*Flavisolibacter_ ginsengisoli*	*Nitrospira_sp*	*bacterium_Ellin504*
D	0.02 ± 0.03 a	0.01 ± 0.02 b	0.01 ± 0.03 a	0.03 ± 0.02 a
H	0.05 ± 0.04 a	0.05 ± 0.04 a	0.05 ± 0.05 a	0.02 ± 0.04 a

Species (%)	*Actinoallomurus _sp*	*Nocardia_ nova*	*Dyella_ marensis*	*Ralstonia_ pickettii*	*Roseisolibact er_agri*
D	0.08 ± 0.15 a	0.28 ± 0.28 a	0.11 ± 0.92 a	0.96 ± 1.81 a	0.00 ± 0.00 a
H	0.00 ± 0.00 a	0.08 ± 0.07 a	0.05 ± 0.06 a	0.08 ± 0.17 a	0.12 ± 0.16 a

Values are presented as mean ± standard deviation (SD). Different lowercase letters within the same column indicate significant differences between treatments ($p < 0.05$, t-test). D = Diseased, H = Healthy. Species listed are those identified as key contributors by random forest analysis. (n=9).
