# Supplementary material for: Rhizosphere microecological characteristics associated with tobacco root-knot nematode disease
Source: Front Microbiol. 2026 Jul 16;17:1874628. doi: 10.3389/fmicb.2026.1874628 (PMC13426051; doi:10.3389/fmicb.2026.1874628)
Supplement: Supplementary file 2 [file Table_1.DOCX]

**SUPPLEMENTARY INFORMATION**

**Rhizosphere Microecological Characteristics Associated with Tobacco Root-Knot Nematode Disease**

**……**

**Tables: 15**

**Figures: 4**

**List of supporting information**

**Table**

**Table S1 Statistical summary of data preprocessing and quality control.**

**Table S2 Comparison of rhizosphere soil indicators between healthy and diseased tobacco plants based on a linear mixed model (LMM).**

**Table S3 Comparison of rhizosphere soil alpha diversity between healthy and diseased tobacco plants based on a linear mixed model (LMM).**

**Table S4 Spearman correlation analysis of bacterial and fungal taxonomic groups.**

**Table S5 Comparison of soil chemical properties between healthy (H) and diseased (D) tobacco rhizosphere soil**

**Table S6 Comparison of soil enzyme activities in the rhizosphere soil of healthy (H) and diseased (D) tobacco plants**

**Table S7 Comparison of alpha diversity indices in rhizosphere soil between healthy (H) and diseased (D) tobacco plants**

**Table S8 Comparison of bacterial phylum relative abundances in rhizosphere soil between healthy (H) and diseased (D) tobacco plants**

**Table S9 Comparison of bacterial genus relative abundances in rhizosphere soil between healthy (H) and diseased (D) tobacco plants**

**Table S10 Comparison of bacterial species relative abundances in rhizosphere soil between healthy (H) and diseased (D) tobacco plants**

**Table S11 Comparison of fungal phylum relative abundances in rhizosphere soil between healthy (H) and diseased (D) tobacco plants**

**Table S12 Comparison of top 10 fungal genera relative abundances in rhizosphere soil between healthy (H) and diseased (D) tobacco plants**

**Table S13 Comparison of top 10 fungal species relative abundances in rhizosphere soil between healthy (H) and diseased (D) tobacco plants**

**Table S14 Comparison of indicator fungal species relative abundances in rhizosphere soil between healthy (H) and diseased (D) tobacco plants**

**Table S15 Comparison of indicator bacterial species relative abundances in rhizosphere soil between healthy (H) and diseased (D) tobacco plants**

**Figure**

**Figure S1. Analysis of soil physicochemical properties and enzyme activities in the rhizosphere soil of healthy (H) and diseased (D) tobacco plants.**

**Figure S2. Rarefaction curves based on Good’s coverage for bacterial (A) and fungal (B) communities in the diseased (D) and healthy (H) groups**

**Figure S3. OTU-level PERMANOVA of rhizosphere soil microbial communities in healthy and diseased tobacco plants from different plots.**

**Figure S4. Random forest analysis identifying indicator species in the rhizosphere soil of healthy (H) and diseased (D) tobacco plants.**

**
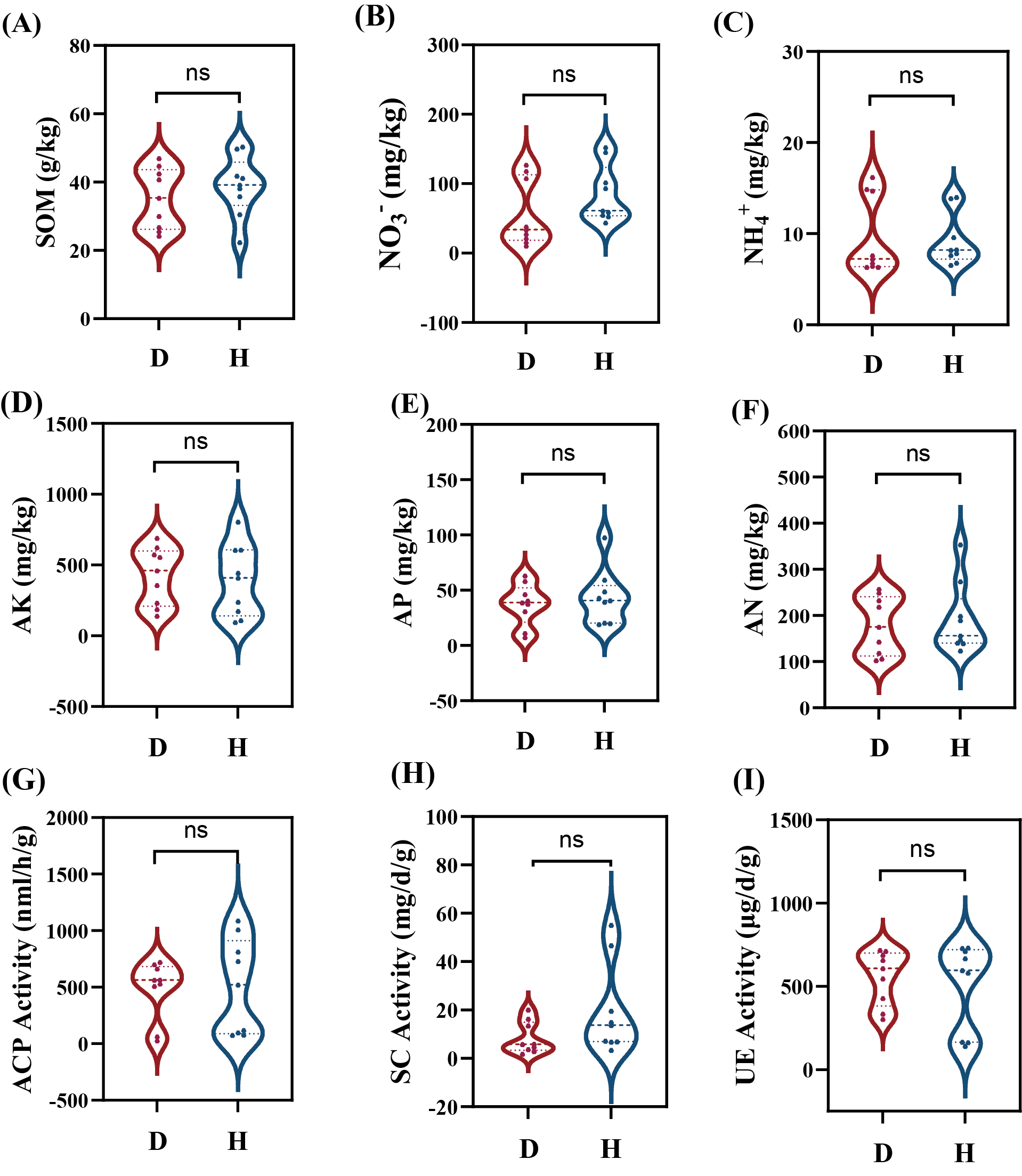
**

**Figure S2.** Analysis of soil physicochemical properties and enzyme activities in the rhizosphere soil of healthy (H) and diseased (D) tobacco plants. (A) Soil organic matter (SOM); (B) Nitrate nitrogen (NO_3_^−^); (C) Ammonium nitrogen (NH_4_^+^); (D) Available potassium (AK); (E) Available phosphorus (AP); (F) Available nitrogen (AN); (G) Acid phosphatase (ACP) activity; (H) Sucrase (SC) activity; (I) Urease (UE) activity. Statistically significant differences are indicated by asterisks (linear mixed model analysis): * *p* < 0.05; “ns” indicates not significant (n = 9).


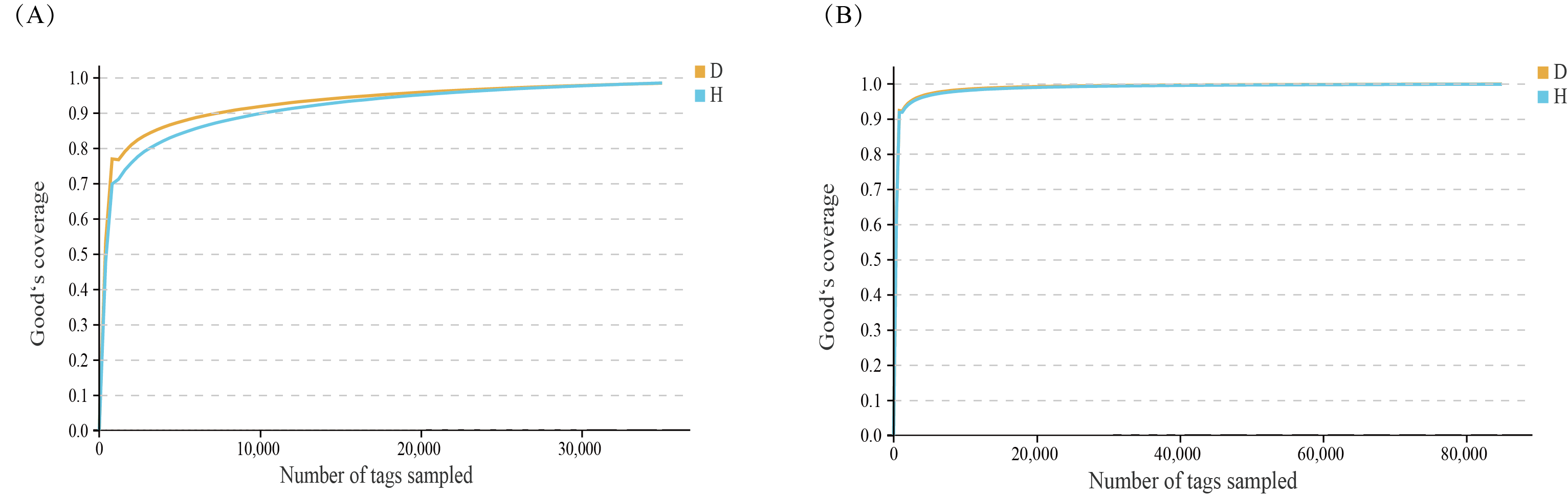


**Figure S2.** Rarefaction curves based on Good’s coverage for bacterial (A) and fungal (B) communities in the diseased (D) and healthy (H) groups (n = 9).


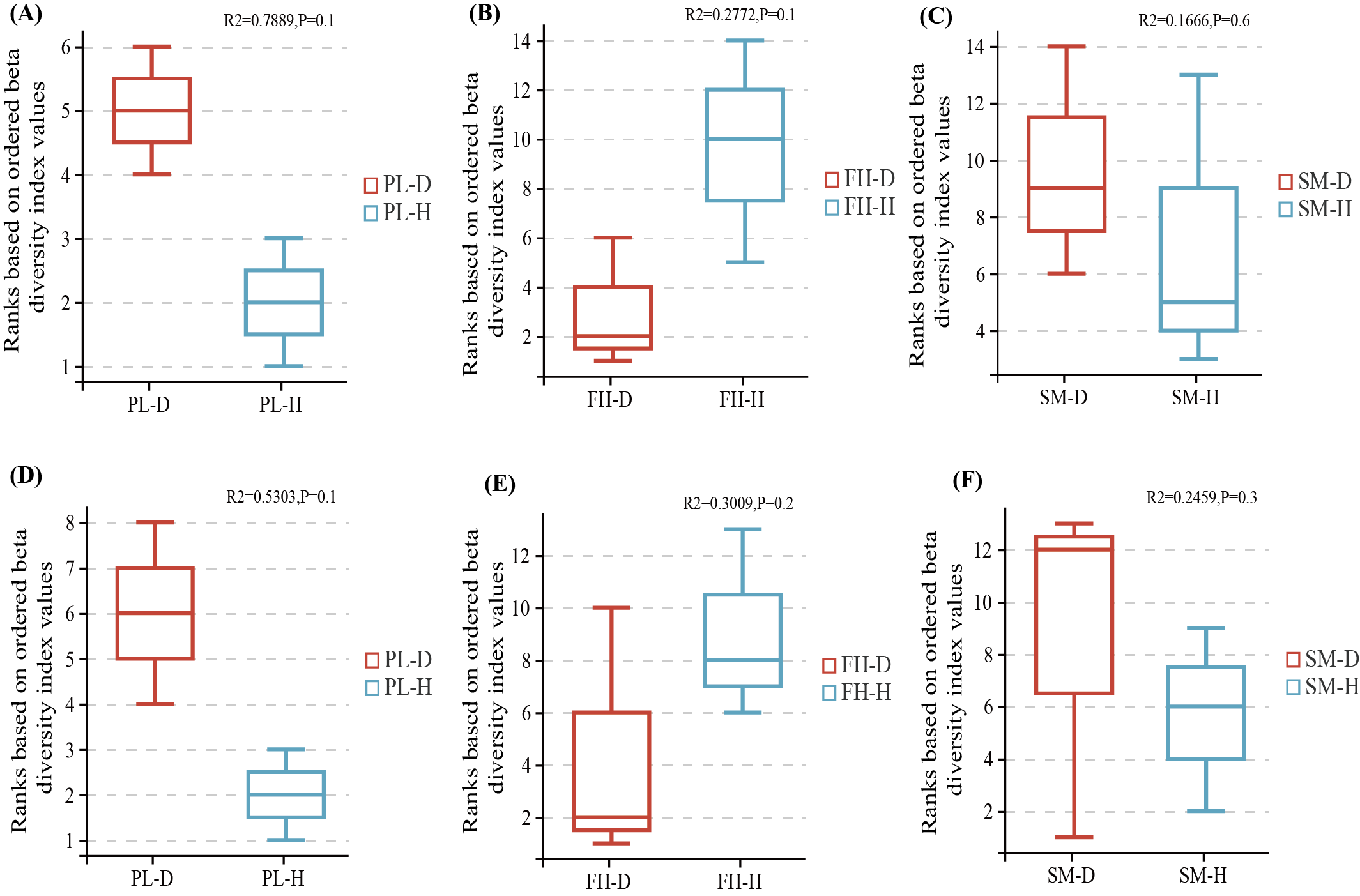


**Figure S3.** OTU-level PERMANOVA of rhizosphere soil microbial communities in healthy and diseased tobacco plants from different plots. Panels (A), (B), and (C) represent bacterial communities; panels (D), (E), and (F) represent fungal communities. PL, Fenghe Town, Xundian County, Kunming; SM, Aziying Township, Songming County, Kunming; D, diseased; H, healthy. Boxplots show the distribution of beta diversity rankings for each group. PERMANOVA (Adonis) results are shown above each panel. R^2^ represents the proportion of variance in microbial community dissimilarity explained by the treatment (healthy vs. diseased), ranging from 0 to 1, with higher values indicating greater treatment effects; P represents the significance level from permutation testing (*p* < 0.05 indicates a statistically significant difference in the community structure between healthy and diseased groups) (n = 3).


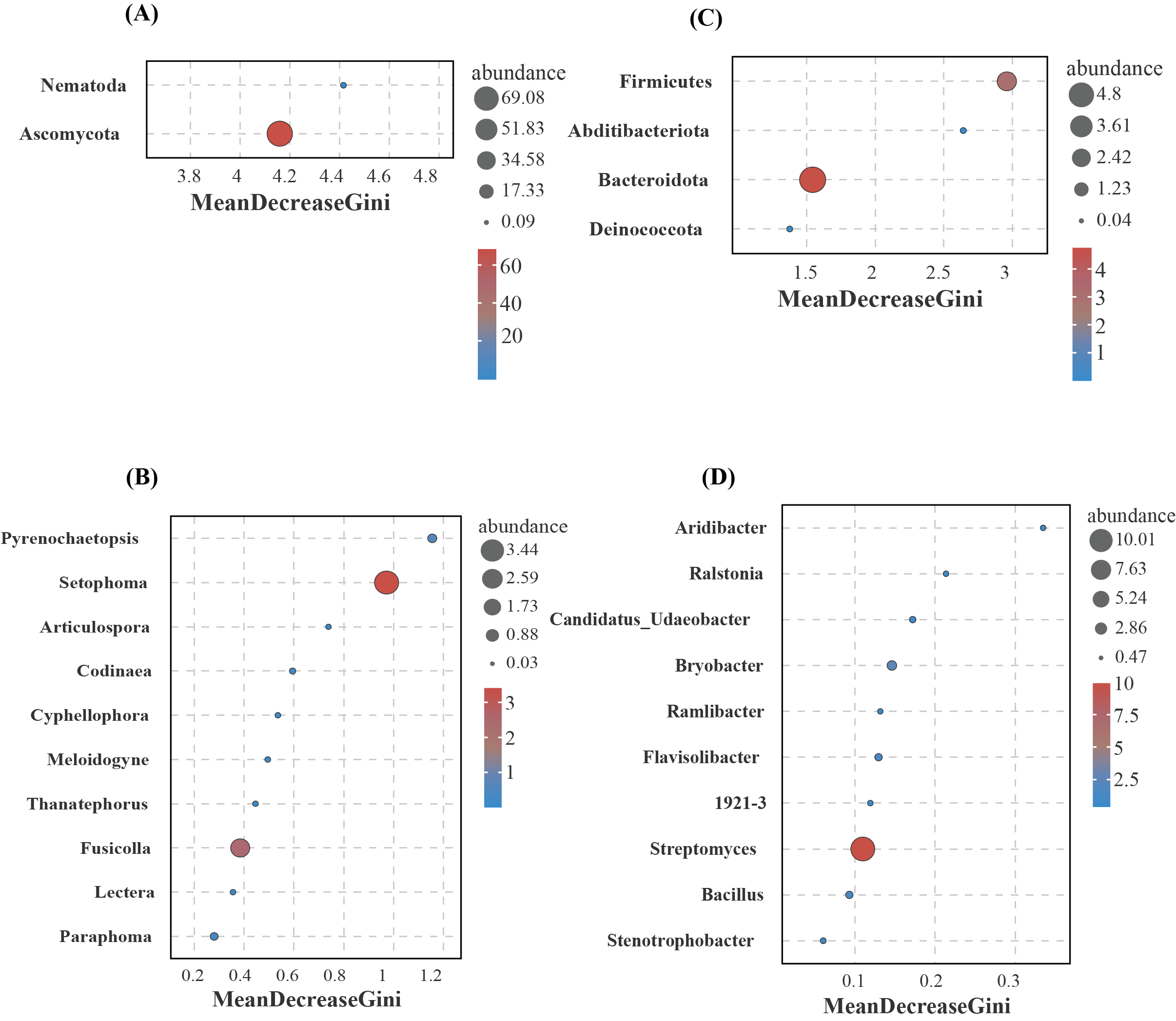


**Figure S4.** Random forest analysis identifying indicator species in the rhizosphere soil of healthy (H) and diseased (D) tobacco plants. (A) Fungal kingdom level; (B) Fungal genus level; (C) Bacterial kingdom level; (D) Bacterial genus level. The size of the circle and depth of the color indicate the magnitude of the abundance (n = 9).

**Table S1 Statistical summary of data preprocessing and quality control.**

| **Sample Name** | | **Raw Reads** | **Clean Reads** | **Raw Tags** | **Clean Tags** | **Chimera** | **Effective Tags** | **Effective Ratio (%)** |
| --- | --- | --- | --- | --- | --- | --- | --- | --- |
| D | FH-D-1 | 126,741 | 126,525 | 124,608 | 123,825 | 30,228 | 93,597 | 73.85 |
|  | FH-D-2 | 128,611 | 128,415 | 126,619 | 125,895 | 37,054 | 88,841 | 69.08 |
|  | FH-D-3 | 135,681 | 135,471 | 133,430 | 132,690 | 35,463 | 97,227 | 71.66 |
|  | FH-H-1 | 125,509 | 125,328 | 123,345 | 122,624 | 36,815 | 85,809 | 68.37 |
|  | FH-H-2 | 136,336 | 136,139 | 133,997 | 133,205 | 40,570 | 92,635 | 67.95 |
|  | FH-H-3 | 126,644 | 126,486 | 124,427 | 123,552 | 38,388 | 85,164 | 67.25 |
|  | PL-D-1 | 124,273 | 124,110 | 122,278 | 121,636 | 39,753 | 81,883 | 65.89 |
|  | PL-D-2 | 121,447 | 121,329 | 119,683 | 115,375 | 31,741 | 83,634 | 68.86 |
|  | PL-D-3 | 130,308 | 130,119 | 128,141 | 127,361 | 38,946 | 88,415 | 67.85 |
| H | PL-H-1 | 136,147 | 135,948 | 133,783 | 132,997 | 40,204 | 92,793 | 68.16 |
|  | PL-H-2 | 131,540 | 131,369 | 129,161 | 128,483 | 40,574 | 87,909 | 66.83 |
|  | PL-H-3 | 123,384 | 123,217 | 121,343 | 120,696 | 38,665 | 82,031 | 66.48 |
|  | SM-D-1 | 127,220 | 127,025 | 124,744 | 123,985 | 39,265 | 84,720 | 66.59 |
|  | SM-D-2 | 128,700 | 128,508 | 126,377 | 125,665 | 40,654 | 85,011 | 66.05 |
|  | SM-D-3 | 128,301 | 128,117 | 125,657 | 124,860 | 39,756 | 85,104 | 66.33 |
|  | SM-H-1 | 136,011 | 135,844 | 133,595 | 132,769 | 43,921 | 88,848 | 65.32 |
|  | SM-H-2 | 136,321 | 136,098 | 134,078 | 133,234 | 41,080 | 92,154 | 67.60 |
|  | SM-H-3 | 134,837 | 134,617 | 132,427 | 131,591 | 41,005 | 90,586 | 67.18 |

For each sample, we recorded the following metrics: the number of raw paired-end reads (Raw PE); the number of high-quality paired-end reads after quality filtering (Clean PE); the number of tags initially obtained after merging overlapping reads (Raw Tags); the number of high-quality tags after quality filtering (Clean Tags); the number of chimeric tags detected during OTU clustering (Chimera); and the number of high-quality tags remaining after chimera removal, which were used as effective tags for downstream analysis (Effective Tags). We also calculated the effective ratio, defined as the percentage of raw paired-end reads that remained effective tags (Effective Ratio, %).

**Table S2 Comparison of rhizosphere soil indicators between healthy and diseased tobacco plants based on a linear mixed model (LMM).**

| **Indicator** | **H** | **D** | **Estimate** | **p-value** | **ICC** |
| --- | --- | --- | --- | --- | --- |
| **pH** | **5.394** | **4.983** | **-0.411** | **0.0092** | **0.067** |
| NH₄⁺ (mg/kg) | 9.207 | 9.621 | 0.414 | 0.6918 | 0.717 |
| NO₃⁻ (mg/kg) | 85.015 | 55.504 | -29.511 | 0.1333 | 0.243 |
| AN (mg/kg) | 191.551 | 178.272 | -13.279 | 0.6557 | 0.281 |
| SOM (g/kg) | 38.790 | 35.214 | -3.577 | 0.402 | 0.000 |
| AP (mg/kg) | 43.269 | 37.157 | -6.112 | 0.4763 | 0.429 |
| AK (mg/kg) | 387.894 | 424.661 | 36.767 | 0.4976 | 0.814 |
| **CAT (cmol/kg)** | **391.928** | **235.853** | **-156.075** | **0.0164** | **0.248** |
| ACP (nmml/h/g) | 503.798 | 482.886 | -20.912 | 0.8925 | 0.209 |
| SC (mg/d/g) | 19.443 | 8.364 | -11.079 | 0.1072 | 0.042 |
| UE (μg/g/h) | 499.043 | 555.568 | 56.525 | 0.4339 | 0.610 |

H, healthy group; D, diseased group; Estimate, estimated treatment effect (difference between diseased and healthy groups); p-value, significance level; ICC, intraclass correlation coefficient, indicating the proportion of total variance attributable to site differences. Significant p-values (p < 0.05) are indicated in bold (n = 9).

**Table S3 Comparison of rhizosphere soil alpha diversity between healthy and diseased tobacco plants based on a linear mixed model (LMM).**

| **Indicator** | | **H** | **D** | **Estimate** | **p-value** | **ICC** |
| --- | --- | --- | --- | --- | --- | --- |
| 16S | **sobs** | **3180.333** | **2696.667** | **-483.667** | **0.034** | **0.000** |
|  | **shannon** | **8.691** | **7.775** | **-0.916** | **0.021** | **0.132** |
|  | simpson | 0.980 | 0.961 | -0.020 | 0.142 | 0.272 |
|  | **chao** | **3274.381** | **2804.395** | **-469.986** | **0.038** | **0.000** |
| ITS | **sobs** | **1067.667** | **911.556** | **-156.111** | **0.015** | **0.235** |
|  | shannon | 5.746 | 5.747 | 0.001 | 0.998 | 0.141 |
|  | simpson | 0.930 | 0.935 | 0.006 | 0.748 | 0.233 |
|  | **chao** | **1218.129** | **1051.327** | **-166.802** | **0.033** | **0.246** |

H, healthy group; D, diseased group; Estimate, estimated treatment effect (difference between diseased and healthy groups); p-value, significance level; ICC, intraclass correlation coefficient, indicating the proportion of total variance attributable to site differences. Significant p-values (p < 0.05) are indicated in bold (n = 9).

**Table S4 Spearman correlation analysis of bacterial and fungal taxonomic groups.**

|  | Ascomycota | *Fusicolla* | *Fusicolla_acetilerea* |
| --- | --- | --- | --- |
| Bacteroidota | 0.310 | 0.546^*^ | 0.546^*^ |
| *Flavisolibacter* | 0.360 | 0.703^**^ | 0.717^**^ |

*Indicates a significant correlation (*p* < 0.05), ** indicates an extremely significant correlation (*p* < 0.01) (n = 9).

**Table S5 Comparison of soil chemical properties between healthy (H) and diseased (D) tobacco rhizosphere soil**

| **Indicator** | **pH** | **NH₄⁺ (mg/kg)** | **NO₃⁻ (mg/kg)** | **AN (mg/kg)** |
| --- | --- | --- | --- | --- |
| **D** | 4.98 ± 0.23 b | 9.62 ± 4.28 a | 55.50 ± 47.65 a | 178.27 ± 62.75 a |
| **H** | 5.39 ± 0.37 a | 9.21 ± 2.83 a | 85.02 ± 40.94 a | 191.55 ± 76.01 a |

| **Indicator** | **AP (mg/kg)** | **AK (mg/kg)** | **SOM (g/kg)** |
| --- | --- | --- | --- |
| **D** | 37.16 ± 18.77 a | 424.66 ± 202.99 a | 35.21 ± 8.85 a |
| **H** | 43.27 ± 24.81 a | 387.89 ± 251.48 a | 38.79 ± 8.78 a |

Values are presented as mean ± standard deviation (SD). Different lowercase letters within the same column indicate significant differences between treatments (p < 0.05, t-test). D = Diseased, H = Healthy. NH₄⁺ = ammonium nitrogen, NO₃⁻ = nitrate nitrogen, AN = alkaline hydrolysis nitrogen, AP = available phosphorus, AK = available potassium, SOM = soil organic matter. (n=9).

**Table S6 Comparison of soil enzyme activities in the rhizosphere soil of healthy (H) and diseased (D) tobacco plants**

| **Indicator** | **CAT (cmol/kg)** | **ACP (nmml/h/g)** | **SC (mg/d/g)** | **UE (μg/g/h)** |
| --- | --- | --- | --- | --- |
| **D** | 235.85 ± 55.30 b | 482.89 ± 259.06 a | 8.36 ± 6.61 a | 555.57 ± 162.09 a |
| **H** | 391.93 ± 196.05 a | 503.80 ± 421.21 a | 19.44 ± 18.69 a | 499.04 ± 261.84 a |

Values are presented as mean ± standard deviation (SD). Different lowercase letters within the same column indicate significant differences between treatments (p < 0.05, t-test). D = Diseased, H = Healthy. CAT: Catalase； SC: Sucrase; ACP: Acid phosphatase; UE: Urease. (n=9).

**Table S7 Comparison of alpha diversity indices in rhizosphere soil between healthy (H) and diseased (D) tobacco plants**

| **Index** | | **Sobs** | **Shannon** | **Simpson** | **Chao1** |
| --- | --- | --- | --- | --- | --- |
| 16S | D | 2696.67 ± 452.88 b | 7.78 ± 1.05 b | 0.96 ± 0.04 a | 2804.40 ± 461.43 b |
|  | H | 3180.33 ± 428.72 a | 8.69 ± 0.51 a | 0.98 ± 0.02 a | 3274.38 ± 416.98 a |
| ITS | D | 911.56 ± 171.99 b | 5.75 ± 0.90 a | 0.94 ± 0.04 a | 1051.33 ± 222.60 a |
|  | H | 1067.67 ± 101.17 a | 5.75 ± 0.81 a | 0.93 ± 0.04 a | 1218.13 ± 104.61 a |

Values are presented as mean ± standard deviation (SD). Different lowercase letters within the same column indicate significant differences between treatments (p < 0.05, t-test). D = Diseased, H = Healthy. (n=9).

**Table S8 Comparison of bacterial phylum relative abundances in rhizosphere soil between healthy (H) and diseased (D) tobacco plants**

| **Phylum(%)** | **Proteobacteria** | **Acidobacteriota** | **Actinobacteriota** | **Chloroflexi** | **Planctomycetota** |
| --- | --- | --- | --- | --- | --- |
| D | 19.89 ± 8.48 a | 11.51 ± 3.91 a | 26.58 ± 14.03 a | 10.31 ± 3.29 a | 5.36 ± 2.41 a |
| H | 22.10 ± 4.66 a | 15.29 ± 4.93 a | 17.57 ± 10.90 a | 9.22 ± 3.82 a | 6.74 ± 1.60 a |

| **Phylum(%)** | **Gemmatimonadota** | **Bacteroidota** | **Firmicutes** | **Patescibacteria** | **Verrucomicrobiota** |
| --- | --- | --- | --- | --- | --- |
| D | 5.01 ± 1.63 a | 2.89 ± 1.66 b | 4.97 ± 5.37 a | 2.45 ± 1.71 a | 2.27 ± 1.62 a |
| H | 5.52 ± 1.75 a | 6.70 ± 4.07 a | 1.43 ± 0.71 a | 2.64 ± 1.04 a | 2.28 ± 1.21 a |

Values are presented as mean ± standard deviation (SD). Different lowercase letters within the same column indicate significant differences between treatments (p < 0.05, t-test). D = Diseased, H = Healthy. Only the top 10 phyla by average relative abundance are shown. Phylum names follow current taxonomic nomenclature. (n=9).

**Table S9 Comparison of bacterial genus relative abundances in rhizosphere soil between healthy (H) and diseased (D) tobacco plants**

| **Genus(%)** | ***Sphingomonas*** | ***Bryobacter*** | ***Streptomyces*** | ***Gemmatimonas*** | ***Flavisolibacter*** |
| --- | --- | --- | --- | --- | --- |
| D | 8.59 ± 4.06 a | 2.04 ± 1.37 a | 13.94 ± 13.42 a | 2.12 ± 1.04 a | 0.61 ± 0.76 b |
| H | 9.17 ± 3.09 a | 3.16 ± 1.15 a | 6.08 ± 8.62 a | 2.43 ± 1.22 a | 2.30 ± 1.52 a |

| **Genus(%)** | ***Bacillus*** | ***Candidatus_Udaeobacter*** | ***JG30a-KF-32*** | ***Bradyrhizobium*** | ***Catenulispora*** |
| --- | --- | --- | --- | --- | --- |
| D | 2.51 ± 4.20 a | 1.38 ± 1.29 a | 0.95 ± 0.50 a | 0.75 ± 0.33 a | 0.81 ± 0.37 a |
| H | 0.39 ± 0.38 a | 0.61 ± 0.91 a | 0.76 ± 1.34 a | 0.87 ± 0.49 a | 0.80 ± 0.96 a |

Values are presented as mean ± standard deviation (SD). Different lowercase letters within the same column indicate significant differences between treatments (p < 0.05, t-test). D = Diseased, H = Healthy. Only the top 10 genera by average relative abundance are shown. (n=9).

**Table S10 Comparison of bacterial species relative abundances in rhizosphere soil between healthy (H) and diseased (D) tobacco plants**

| **Species(%)** | ***Ralstonia_***  ***pickettii*** | ***Catenulispora_***  ***sp*** | ***Paraburkholderia_caledonica*** | ***Niastella_koreensis_GR20-10*** | ***Catenulispora_***  ***acidiphila_DSM_44928*** |
| --- | --- | --- | --- | --- | --- |
| D | 0.96 ± 1.81 a | 0.56 ± 0.33 a | 0.73 ± 1.27 a | 0.38 ± 0.44 a | 0.23 ± 0.14 a |
| H | 0.08 ± 0.17 a | 0.39 ± 0.36 a | 0.31 ± 0.49 a | 0.29 ± 0.68 a | 0.38 ± 0.66 a |

| **Species (%)** | ***Sphingomonas_***  ***mali*** | ***Nocardia_***  ***nova*** | ***Amycolatopsis_***  ***mediterranei_S699*** | ***Kribbella_***  ***karoonensis*** | ***Romboutsia_***  ***ilealis*** |
| --- | --- | --- | --- | --- | --- |
| D | 0.18 ± 0.09 a | 0.28 ± 0.28 a | 0.16 ± 0.13 a | 0.12 ± 0.12 a | 0.09 ± 0.06 a |
| H | 0.27 ± 0.15 a | 0.08 ± 0.07 a | 0.16 ± 0.19 a | 0.08 ± 0.08 a | 0.08 ± 0.06 a |

Values are presented as mean ± standard deviation (SD). Different lowercase letters within the same column indicate significant differences between treatments (p < 0.05, t-test). D = Diseased, H = Healthy. Only the top 10 species by average relative abundance are shown. (n=9).

**Table S11 Comparison of fungal phylum relative abundances in rhizosphere soil between healthy (H) and diseased (D) tobacco plants**

| **Phylum (%)** | **Basidiomycota** | **Mucoromycota** | **Ascomycota** | **Anthophyta** | **Chlorophyta** |
| --- | --- | --- | --- | --- | --- |
| H | 7.60 ± 1.73 a | 2.65 ± 1.35 a | 75.92 ± 2.91 a | 1.80 ± 0.40 a | 4.44 ± 1.76 a |
| D | 13.73 ± 1.73 a | 6.55 ± 1.35 a | 62.23 ± 2.91 b | 7.08 ± 0.40 a | 2.45 ± 1.76 a |

| **Phylum (%)** | **Mortierellomycota** | **Chytridiomycota** | **Glomeromycota** | **Rozellomycota** | **Ciliophora** |
| --- | --- | --- | --- | --- | --- |
| H | 1.66 ± 0.51 a | 1.77 ± 0.67 a | 0.13 ± 0.02 a | 0.78 ± 0.63 a | 0.87 ± 0.39 a |
| D | 2.79 ± 0.51 a | 0.91 ± 0.67 a | 1.20 ± 0.02 a | 0.51 ± 0.63 a | 0.10 ± 0.39 a |

Values are presented as mean ± standard deviation (SD). Different lowercase letters within the same column indicate significant differences between treatments (p < 0.05, t-test). D = Diseased, H = Healthy. Only the top 10 phyla by average relative abundance are shown. (n=9).

**Table S12 Comparison of top 10 fungal genera relative abundances in rhizosphere soil between healthy (H) and diseased (D) tobacco plants**

| **Genus (%)** | ***Nicotiana*** | ***Saitozyma*** | ***Fusarium*** | ***Setophoma*** | ***Trichoderma*** |
| --- | --- | --- | --- | --- | --- |
| D | 6.19 ± 8.04 a | 4.50 ± 2.52 a | 11.42 ± 7.11 a | 4.10 ± 3.21 a | 1.30 ± 1.47 a |
| H | 1.41 ± 1.26 a | 2.91 ± 2.49 a | 19.87 ± 11.17 a | 2.78 ± 5.46 a | 4.36 ± 8.03 a |

| **Genus (%)** | ***Fusicolla*** | ***Mortierella*** | ***Talaromyces*** | ***Rhizopus*** | ***Coniochaeta*** |
| --- | --- | --- | --- | --- | --- |
| D | 0.57 ± 0.50 a | 2.79 ± 2.88 a | 3.94 ± 7.75 a | 4.01 ± 10.79 a | 2.19 ± 1.37 a |
| H | 4.47 ± 4.36 b | 1.66 ± 1.54 a | 0.48 ± 0.28 a | 0.02 ± 0.02 a | 1.34 ± 1.10 a |

Values are presented as mean ± standard deviation (SD). Different lowercase letters within the same column indicate significant differences between treatments (p < 0.05, t-test). D = Diseased, H = Healthy. Only the top 10 genera by average relative abundance are shown. (n=9).

**Table S13 Comparison of top 10 fungal species relative abundances in rhizosphere soil between healthy (H) and diseased (D) tobacco plants**

| **Species (%)** | **Saitozyma_**  **podzolica** | **Setophoma_**  **terrestris** | **Fusarium_**  **solani** | **Fusicola_**  **acetilerea** | **Rhizopus_**  **microsporus** |
| --- | --- | --- | --- | --- | --- |
| H | 2.66 ± 2.65 a | 2.78 ± 5.46 a | 6.34 ± 9.05 a | 4.28 ± 4.19 a | 0.02 ± 0.02 a |
| D | 4.45 ± 2.52 a | 4.10 ± 3.21 a | 1.02 ± 0.78 a | 0.52 ± 0.50 b | 4.00 ± 10.79 a |

| **Species (%)** | **Solicoceozyma_**  **fuscescens** | **Mortierella_**  **elongata** | **Spiromastix_**  **warcupii** | **Nicotiana_**  **occidentalis** | **Podospora_**  **communis** |
| --- | --- | --- | --- | --- | --- |
| H | 1.53 ± 2.13 a | 0.84 ± 1.06 a | 0.49 ± 0.64 a | 0.43 ± 0.44 a | 1.28 ± 2.44 a |
| D | 1.91 ± 1.27 a | 1.66 ± 2.63 a | 1.39 ± 2.10 a | 1.24 ± 1.25 a | 0.15 ± 0.17 a |

Values are presented as mean ± standard deviation (SD). Different lowercase letters within the same column indicate significant differences between treatments (p < 0.05, t-test). D = Diseased, H = Healthy. Only the top 10 species by average relative abundance are shown. (n=9).

**Table S14 Comparison of indicator fungal species relative abundances in rhizosphere soil between healthy (H) and diseased (D) tobacco plants**

| **Species (%)** | ***Fusicolla_***  ***acetilerea*** | ***Pyrenochaetopsis_***  ***leptospora*** | ***Setophoma_***  ***terrestris*** | ***Codinaea_***  ***acaciae*** |
| --- | --- | --- | --- | --- |
| D | 0.52 ± 0.50 b | 0.08 ± 0.06 a | 4.10 ± 3.21 a | 0.32 ± 0.61 a |
| H | 4.28 ± 4.19 a | 1.26 ± 1.67 a | 2.78 ± 5.46 a | 0.03 ± 0.03 a |

| **Species (%)** | ***Meloidogyne_***  ***incognita*** | ***Thanatephorus_***  ***cucumeris*** | ***Metarhizium_***  ***marquandii*** | ***Fusarium_***  ***cuneirostrum*** |
| --- | --- | --- | --- | --- |
| D | 0.15 ± 0.26 a | 0.10 ± 0.10 a | 0.07 ± 0.05 a | 0.01 ± 0.01 b |
| H | 0.01 ± 0.01 a | 0.02 ± 0.01 b | 0.02 ± 0.02 a | 0.08 ± 0.09 a |

Values are presented as mean ± standard deviation (SD). Different lowercase letters within the same column indicate significant differences between treatments (p < 0.05, t-test). D = Diseased, H = Healthy. Species listed are those identified as key contributors by random forest analysis. (n=9).

**Table S15 Comparison of indicator bacterial species relative abundances in rhizosphere soil between healthy (H) and diseased (D) tobacco plants**

| **Species (%)** | ***Rhizobium_***  ***mesosinicum*** | ***Flavisolibacter_***  ***ginsengisoli*** | ***Nitrospira_sp*** | ***bacterium_Ellin504*** |
| --- | --- | --- | --- | --- |
| D | 0.02 ± 0.03 a | 0.01 ± 0.02 b | 0.01 ± 0.03 a | 0.03 ± 0.02 a |
| H | 0.05 ± 0.04 a | 0.05 ± 0.04 a | 0.05 ± 0.05 a | 0.02 ± 0.04 a |

| **Species (%)** | ***Actinoallomurus_sp*** | ***Nocardia_***  ***nova*** | ***Dyella_***  ***marensis*** | ***Ralstonia_***  ***pickettii*** | ***Roseisolibacter_agri*** |
| --- | --- | --- | --- | --- | --- |
| D | 0.08 ± 0.15 a | 0.28 ± 0.28 a | 0.11 ± 0.92 a | 0.96 ± 1.81 a | 0.00 ± 0.00 a |
| H | 0.00 ± 0.00 a | 0.08 ± 0.07 a | 0.05 ± 0.06 a | 0.08 ± 0.17 a | 0.12 ± 0.16 a |

Values are presented as mean ± standard deviation (SD). Different lowercase letters within the same column indicate significant differences between treatments (p < 0.05, t-test). D = Diseased, H = Healthy. Species listed are those identified as key contributors by random forest analysis. (n=9).
